# SHP2 regulates skeletal cell fate by modifying SOX9 expression and transcriptional activity

**DOI:** 10.1038/s41413-018-0013-z

**Published:** 2018-04-06

**Authors:** Chunlin Zuo, Lijun Wang, Raghavendra M. Kamalesh, Margot E. Bowen, Douglas C. Moore, Mark S. Dooner, Anthony M. Reginato, Qian Wu, Christoph Schorl, Yueming Song, Matthew L. Warman, Benjamin G. Neel, Michael G. Ehrlich, Wentian Yang

**Affiliations:** 1 Department of Orthopaedics, Brown University Alpert Medical School and Rhode Island Hospital, Providence, RI 02903 USA; 2 Orthopaedic Research Laboratories and Howard Hughes Medical Institute, Boston Children’s Hospital and Department of Genetics, Harvard Medical School, Boston, MA 02115 USA; 3 Division of Hematology and Oncology, Brown University Alpert Medical School and Rhode Island Hospital, Providence, RI 02903 USA; 4 Division of Rheumatology, Brown University Alpert Medical School and Rhode Island Hospital, Providence, RI 02903 USA; 5 Department of Pathology and Laboratory Medicine, University of Connecticut Health Center, Farmington, CT 06030 USA; 6 Department of Molecular and Cell Biology and Biochemistry, Brown University, 70 Ship Street, Providence, RI 02912 USA; 7 Department of Orthopedic Surgery, West China Hospital of Sichuan University, Chengdu, 610041 China; 8 Laura and Issac Perlmutter Cancer Center, NYU Langone Medical Center, New York, NY 10016 USA; 9 Present Address: Department of Endocrinology, the First Affiliated Hospital of Anhui Medical University, Hefei, 230022 China

## Abstract

Chondrocytes and osteoblasts differentiate from a common mesenchymal precursor, the osteochondroprogenitor (OCP), and help build the vertebrate skeleton. The signaling pathways that control lineage commitment for OCPs are incompletely understood. We asked whether the ubiquitously expressed protein-tyrosine phosphatase SHP2 (encoded by *Ptpn11*) affects skeletal lineage commitment by conditionally deleting *Ptpn11* in mouse limb and head mesenchyme using “Cre-loxP”-mediated gene excision. SHP2-deficient mice have increased cartilage mass and deficient ossification, suggesting that SHP2-deficient OCPs become chondrocytes and not osteoblasts. Consistent with these observations, the expression of the master chondrogenic transcription factor SOX9 and its target genes *Acan, Col2a1*, and *Col10a1* were increased in SHP2-deficient chondrocytes, as revealed by gene expression arrays, qRT-PCR, in situ hybridization, and immunostaining. Mechanistic studies demonstrate that SHP2 regulates OCP fate determination via the phosphorylation and SUMOylation of SOX9, mediated at least in part via the PKA signaling pathway. Our data indicate that SHP2 is critical for skeletal cell lineage differentiation and could thus be a pharmacologic target for bone and cartilage regeneration.

## Chondrogenesis: SHP2 regulates skeletal cell fate

The enzyme SHP2 is critical for the differentiation of skeletal cells and could be a pharmacological target for bone and cartilage regeneration. Osteo-chondroprogenitor cells arise from stem cells in the bone marrow and can differentiate into either osteoblasts (bone-producing cells) or chondrocytes (cartilage cells). A team headed by Wentian Yang at Brown University, Rhode Island found that mice deficient in SHP2 had increased cartilage mass and decreased ossification. As a result of SHP2 deficiency, abnormal cartilage growth developed at sites where mineralized bone would normally form. The team’s findings suggest that SHP2-deficient osteo-chondroprogenitor cells become chondrocytes, not osteoblasts. The authors believe that the ability to therapeutically manipulate cell fate by regulating SHP2 activity offers a new means of controlling cartilage formation in patients with various cartilage-related disorders, including tumors and osteoarthritis.

## Introduction

Vertebrate skeletal development occurs through intramembranous and endochondral ossification. Intramembranous ossification involves the direct differentiation of mesenchymal stem cells into osteoblasts and is responsible for the ossification of cranial bones and for appositional bone growth.^1,2^ Endochondral ossification requires the formation of cartilaginous anlagen and their subsequent replacement by osteoblasts, and contributes to longitudinal bone growth.^3,4^ During endochondral ossification, mesenchymal cells condense and then differentiate into early proliferating chondrocytes, which undergo further differentiation to establish a cartilage growth plate. Cells within growth plates are organized into distinct zones containing resting, proliferating, pre-hypertrophic, and hypertrophic chondrocytes. Hypertrophic chondrocytes undergo apoptosis and are replaced by osteoblasts or transdifferentiate into osteoblasts, which produce bone.^5–10^

Signaling molecules and transcription factors, including SOX9,^11,12^ β-CATENIN,^13^ and RUNX2,^14,15^ regulate skeletal development. The transcription factor SOX9 is a master regulator of chondrogenesis, essential for chondrocyte specification, proliferation, and early differentiation.^12,16,17^ SOX9 promotes the expression of important chondrocytic genes, including *Col2a1*, *Col10a1*, and *Acan*.^11,16^ Whereas SOX9 is critical for chondroid cell fate determination and chondrocytic differentiation, β-CATENIN is a critical regulator of osteoblast differentiation and osteogenesis. WNT ligand-induced β-CATENIN signaling mediates major events in endochondral and intramembranous bone formation.^18^ Increased abundance of SOX9 or β-CATENIN promote chondrogenesis or osteoblastogenesis, respectively.^13,19–21^

Transcription factor activity can be regulated at the transcriptional and post-translational levels. One post-translational mechanism, SUMOylation, tags proteins with small ubiquitin-like molecules (SUMO)^22,23^ that can alter the biological functions of their targets. Another post-translation regulatory modification is phosphorylation. SOX9 can be SUMOylated and phosphorylated.^24,25^ How SUMOylation is regulated remains elusive, but pathways involving the serine-threonine kinases ERK, ROCK, and PKA have been implicated.^26–30^

SHP2, encoded by *PTPN11*, is a ubiquitously expressed Src homology-2 domain-containing protein-tyrosine phosphatase. SHP2 is required for activation of the RAS/ERK pathway downstream of almost all receptor protein-tyrosine kinases (RTK) and cytokine and integrin receptors.^31,32^ There is evidence that SHP2 plays an important role in skeletal development. Notably, autosomal dominant mutations in *PTPN11* cause Noonan and LEOPARD syndromes (NS and LS, respectively), which feature skeletal manifestations that can include pectus carinatum or pectus excavatum, short stature, and scoliosis.^33,34^ Heterozygous SHP2 loss-of-function (LOF) mutations are responsible for the autosomal dominant disorder metachondromatosis, in which somatic second hit *PTPN11* mutations give rise to enchondromas and exostoses.^35,36^ We and others have demonstrated that inactivation of *Ptpn11* in cells committed to the chondrogenic lineage impairs terminal differentiation to chondrocytes, and inactivation at other sites may promote chondrogenesis instead of osteogenesis.^37,38^ However, the role of SHP2 in modulating cell fate decisions in OCPs remains unexplored.

By utilizing a tissue-specific *Ptpn11* gene ablation approach, we report here that SHP2 deficiency in both limb and head mesenchymal progenitors impairs cartilage, bone and joint development. SHP2 regulates chondrogenesis by modulating the lineage commitment of mesenchymal progenitors and by repressing chondrocytic differentiation, and this regulation is mediated at least in part by influencing the phosphorylation and SUMOylation of SOX9 via the PKA signaling pathway.

## Results

### SHP2 deficiency in limb and head mesenchyme affects skeletogenesis

To investigate the role of SHP2 in limb and head mesenchymal cells during early skeletogenesis, mice carrying *Ptpn11* floxed (*Ptpn11*^*fl/+*^) alleles^39^ were crossed to paired related homeobox 1 *Tg(Prrx1-Cre)*^40^ and *Tg(Prrx1-CreERt2)*^41^ Cre mice to generate *Tg(Prrx1-Cre;Ptpn11*^*fl/+*^*)* (SHP2_Prrx1_CTR), *Tg(Prrx1-Cre;Ptpn11*^*fl/fl*^*)* (SHP2_Prrx1_KO), *Tg(Prrx1-CreERt2;Ptpn11*^*fl/+*^*)* (SHP2_Prrx1_CTR/ER) and *Tg(Prrx1-CreERt2;Ptpn11*^*fl/fl*^*)* (SHP2_Prrx1_KO/ER) mice (Fig. S1a). The *Prrx1* promoter is active both in the undifferentiated mesenchyme of limb buds^42^ and in the periosteum of adult mice.^43^ Therefore, in SHP2_Prrx1_KO and SHP2_Prrx1_KO/ER mice, *Ptpn11* is specifically deleted in PRRX1-expressing mesenchymal osteochondroprogenitors (OCPs) and their progeny. The deletion efficiency of *Ptpn11* floxed alleles in OCPs and their derivatives by *Prrx1-Cre* or *Prrx1-CreERt2* was determined by Western blot analysis, which revealed that SHP2 abundance was reduced by > 80% and > 70% in purified OCPs and their derivatives from SHP2_Prrx1_KO or tamoxifen-treated SHP2_Prrx1_KO/ER mice respectively, compared with those from SHP2_Prrx1_CTR and SHP2_Prrx1_CTR/ER controls (Fig. S1b). SHP2_Prrx1_CTR and SHP2_Prrx1_CTR/ER mice had no discernible phenotype, so subsequent analyses were focused on SHP2_Prrx1_KO and SHP2_Prrx1_KO/ER mice.

**Fig. 1:**
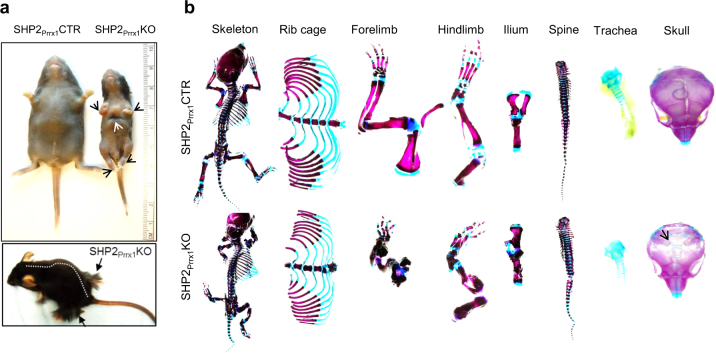
Mice lacking SHP2 in PRRX1-expressing osteochondroprogenitors (OCPs) display skeletal dysplasia and impaired ossification of multiple skeletal elements. **a** Representative photographs of 10-day-old SHP2_Prrx1_CTR and SHP2_Prrx1_KO mice. SHP2_Prrx1_KO mice have skeletal dwarfism, with short and deformed forelimbs and hindlimbs (black arrows), and concave-appearing ribcage (white arrow) (*n* = 5). A few SHP2_Prrx1_KO survivors grew long hairs surrounding both forelimbs and hind limbs (black arrows) (*n* = 3). **b** Alcian blue/Alizarin red-stained skeletal preps of the entire skeleton, rib cage and sternum, forelimbs, hindlimbs, ilium, spine, trachea, and the skull of 7-day-old SHP2_Prrx1_CTR and SHP2_Prrx1_KO mice. Note that SHP2_Prrx1_KO mice have small ribcages, retarded ossification of sternum and skull, short and deformed forelimbs, hindlimbs, trachea, and metatarsal and phalange joint digits (*n* = 3).

SHP2_Prrx1_KO mice were born at the expected Mendelian ratios and they were the same size as the SHP2_Prrx1_CTR littermate controls at birth, on average [(48.8 ± 3.5)mm vs. (49.0 ± 4.2)mm long at P0.5, *P* = 0.96]. By postpartum day 10 (P10) both the controls and knockouts had grown significantly (*P* < 0.001, both comparisons), however, the body length of SHP2_Prrx1_KO mice was only ~75% of SHP2_Prrx1_CTR controls [SHP2_Prrx1_CTR/SHP2_Prrx1_KO: (97.4 ± 5.4)mm vs. (74.4 ± 8.4)mm; *n* = 5, *P* < 0.001]. Most of the mutants died within 3 weeks after birth, likely due to respiratory failure. Other skeletal phenotypes included short and deformed forelimbs and hindlimbs, and pectus anomalies (carinatum or excavatum) (Fig. 1a; S1c). Interestingly, a few of the surviving SHP2_Prrx1_KO mice developed localized hypertrichosis on the forelimbs and hind limbs (Fig. 1a, bottom), which has been reported to be related to increased SOX9 expression in the hair follicle.^44^ Alcian blue and Alizarin Red staining of the skeletons in the mutants revealed split sternums, short appendicular bones and short tracheas (Fig. 1b). Compared with SHP2_Prrx1_CTR mice, the skulls of SHP2_Prrx1_KO mice were incompletely formed, with gross defects in the interparietal, parietal and frontal bones (Fig. 1b). Collectively, these data suggest that SHP2 plays a crucial role in PRRX1-expressing OCPs in both endochondral and intramembranous bone formation.

### SHP2 deficiency in OCPs promotes cell proliferation and chondrocytic differentiation

**Fig. 2:**
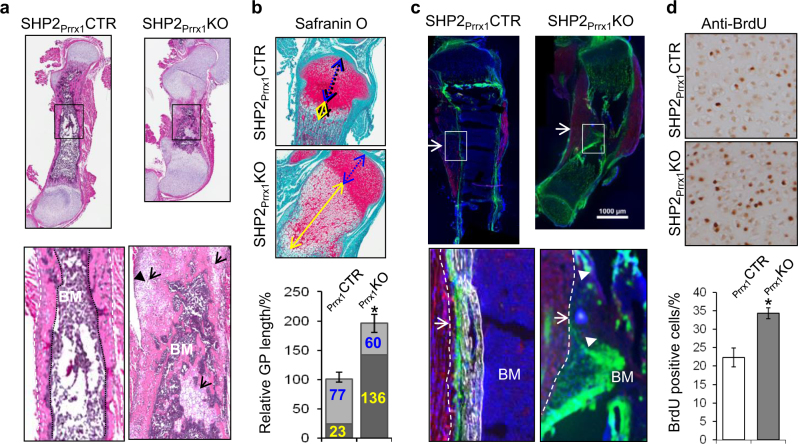
SHP2 deficiency in PRRX1-expressing OCPs delays endochondral ossification and leads to ectopic cartilage formation. **a** Representative images of H&E-stained longitudinal sections of femurs demonstrate impaired ossification of appendicular bones, enhanced chondrogenesis and ectopic cartilage formation in 7-day-old SHP2_Prrx1_KO mice, compared with SHP2_Prrx1_CTR Mice. Bottom panels are enlarged views (×10) of corresponding boxed areas in the top panel showing ectopic chondrocytes in the bone cortex and islands of chondrocytes in the bone marrow (BM, arrow) of SHP2_Prrx1_KO mice (*n* = 4). **b** Images of sagittal sections of proximal tibiae (top), stained with Safranin O/fast green, showing the broad and elongated growth plate cartilage in SHP2_Prrx1_KO mice. Bar graphs (bottom) show 2- and 5-fold increases in height of the epiphyseal cartilage (blue+yellow lines) and the pre-hypertrophic and hypertrophic layers, (yellow line) respectively, in SHP2_Prrx1_KO mice, compared with SHP2_Prrx1_CTR mice (*n* = 4). *P < 0.05 (Student’s *t* test). **c** Fluorescence microscopy of frozen tibia sections demonstrates that PRRX1-expressing cells (GFP^+^) primarily exist as a thin layer (periosteum, arrow) on the surface of the mineralized bone cortex and also appear in the epiphyseal cartilage of 2-day-old SHP2_Prrx1_CTR;R26^mTmG^ reporter mice. By contrast, in age-matched SHP2_Prrx1_KO;R26^mTmG^ mice, there was no mineralized cortical bone, and the GFP+ cells were not restricted to the thin periosteal soft tissue layer along what would have been the cortex (arrows). Instead, GFP^+^ OCPs and their derivatives were scattered in non-mineralized areas where cortical bone should have formed and in cartilage islands within the bone marrow (arrow head) (*n* = 4). Bottom panels are enlarged views (×10) of the corresponding boxed areas in top panels. Dashed lines denote the boundary between muscle and bone. Bone marrow, BM. **d** Images of anti-BrdU staining of proximal tibia sections demonstrate the increase of BrdU^+^ cells in the proliferative zone of SHP2_Prrx1_KO mice, compared to that of SHP2_Prrx1_CTR. Bar graphs at the bottom show quantification of these data from replicates (*n* = 3, *P < 0.01, Student’s *t* test).

To begin to understand how SHP2 in OCPs regulates skeletogenesis, femur and tibia sections from 7-day-old SHP2_Prrx1_CTR and SHP2_Prrx1_KO mice were stained with hematoxylin and eosin (H&E) and Safranin O/fast green. The bones from SHP2_Prrx1_CTR mice appeared normal, with distinct mineralized cortices and trabecular bone and hematopoietic cells occupying the cavity between the organized growth plate cartilage. By contrast, the bones of SHP2_Prrx1_KO mice contained large epiphyseal cartilage masses, but no clearly ossified cortical nor trabecular bone. Instead, substantial numbers of chondrocytes remained in the regions that would have been the diaphyseal cortices of the femurs in SHP2_Prrx1_KO mice, and there were persistent “cartilage islands” scattered throughout the marrow cavity (Fig. 2a). Moreover, the growth plates of SHP2_Prrx1_KO mutants were profoundly affected. Rather than the typical, tightly-organized columns of chondrocytes seen in SHP2_Prrx1_CTR mice, SHP2_Prrx1_KO mutant growth plates were taller and much less organized. The growth plates in the mutant animals had a 2-fold increase in the height of epiphyseal cartilage and a 5-fold increase of the pre-hypertrophic and hypertrophic layers of the growth plate cartilage compared with controls (Fig. 2b, S2a). To rule out the possibility that impaired ossification in SHP2_Prrx1_KO mice was due to the effect of SHP2 deficiency on osteoblast differentiation rather than OCP commitment, we deleted SHP2 in committed osteoblasts by crossing the *Ptpn11* floxed allele to *Tg(Bglap-Cre)*^45^ mice. As *Tg(Bglap-Cre)* is expressed in committed osteoblasts, this deletion differentiates the roles for SHP2 in OCPs and fully differentiated osteoblastic cells. Importantly, *Tg(Bglap-Cre;Ptpn11*^*fl/fl*^*)* mice had normal appearing trabecular and cortical bone at day P0.5 and by 8 weeks old (Fig. S10), which was not the case for *Tg(Prrx1-Cre;Ptpn11*^*fl/fl*^*)* mice. These results strongly suggest that SHP2's major role occurs during OCP commitment to the osteoblast lineage.

SHP2-deficient chondroid cells produce autocrine and paracrine signals.^37^ We therefore sought to determine whether the phenotype we observed in SHP2_Prrx1_KO mice was due to the SHP2-deficient OCPs (i.e., whether it was OCP-autonomous) or whether these OCPs might be influencing other cells. To do so, we performed a cell lineage tracing study by crossing SHP2_Prrx1_CTR and SHP2_Prrx1_KO mice to cell membrane-targeted two-color fluorescent Cre reporter mice *Rosa26*^*mTmG*^ (R26^mTmG^, expressing red fluorescent protein in cells prior to Cre exposure, and green fluorescent protein in Cre-expressing cells and their derivatives). As expected, GFP^+^ OCPs were found in a thin, continuous layer on the surface of mineralized bone cortex (periosteum) and in the epiphyseal cartilage of SHP2_Prrx1_CTR mice (Fig. 2c, left). By contrast, this cell population was scattered throughout what would have been the cortex and bone marrow compartments of the femurs and tibiae in SHP2_Prrx1_KO mice; no mature calcified cortical bone formed in these mice at postnatal day 10 (Fig. 2c, right; S2b). Coupled with their persistent cartilage phenotype, the localization of GFP+ cells to regions that should have been mineralized bone suggests that SHP2 deficiency resulted in the cell-autonomous differentiation of OCPs along the chondrocyte rather than osteoblast lineage. However, the possibility that altered paracrine signaling arising from SHP2-deficient OCPs contributes to the skeletal phenotypes cannot be conclusively excluded.

To ask whether SHP2 deficiency might also affect cell proliferation, as in other types of cells,^46,47^ we administered BrdU to 10-day-old SHP2_Prrx1_CTR and SHP2_Prrx1_KO mice for 4 h prior to euthanasia. Proximal tibia sections from these mice revealed an increase of BrdU-positive cells in the epiphyseal cartilage of SHP2_Prrx1_KO mice (34.4%), compared with SHP2_Prrx1_CTR controls (22.3%) (Fig. 2d). Similar findings were obtained via an in vitro BrdU labeling assay using chondroprogenitors isolated from SHP2_Prrx1_CTR and SHP2_Prrx1_KO mice (Fig. S2c); their viability was, however, comparable, as determined by annexin-V staining and flow cytometry analysis. Taken together, these experiments suggest that cell fate in OCPs is regulated by SHP2, as is the rate of growth plate chondrocyte proliferation and differentiation.

### SHP2 differentially regulates chondrocytic gene expression

**Fig. 3:**
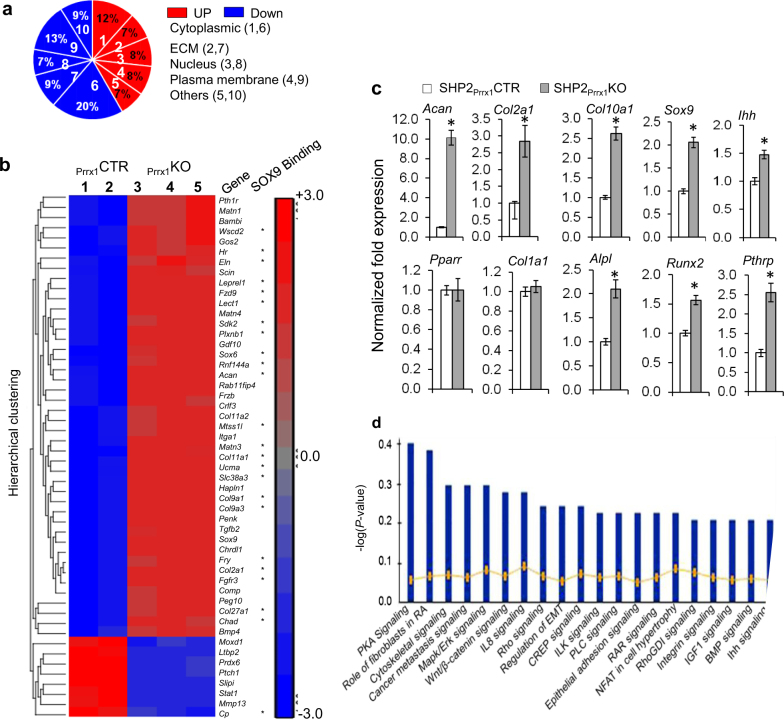
SHP2 negatively regulates chondrocytic gene expression **a** Pie chart demonstrating the clusters of genes that are differentially regulated by SHP2 in OCPs and their derivatives. Cytoplasmic, cytoplasmic proteins, ECM, extracellular matrix proteins; Nucleus, nuclear proteins and transcription factors. **b** Heat map and list of selected chondrogenic genes that increased or decreased in abundance following *Ptpn11* deletion in OCPs and derivatives. Note that most of chondrocytic genes that increased in abundance harbor SOX9 binding sites (asterisks). **c** Bar graphs of qRT-PCR data showing the enhanced expression of *Acan, Col2α1, Col10α1, Sox9, Ihh, Runx2*, and *Pthrp* in OCPs and their derivatives from SHP2_Prrx1_KO mice, compared with SHP2_Prrx1_CTR controls. *PPARγ* and *Col1a1* transcripts are comparable between these animals. Data are presented as the fold changes of mRNA abundance relative to the corresponding controls. All samples are normalized to *Gapdh* (*n* = 3, *P < 0.01, Student’s *t* test). **d** Bar graphs depicting the top 20 cellular signaling pathways predicted to be substantially affected by *Ptpn11* deletion in OCPs and their derivatives by Ingenuity Pathway Analysis. Note that PKA signaling pathway was at the top of all affected signaling pathways.

Given that SHP2 influences OCP cell fate, we next sought to define the genes and pathways that might be involved. To start, we used differential gene expression array analysis to identify transcripts that varied in abundance in SHP2-deficient OCPs and their derivatives, compared to wild-type controls. GFP^+^ OCPs and their derivatives from SHP2_Prrx1_CTR;R26^mTmG^ and SHP2_Prrx1_KO;R26^mTmG^ mice were purified by FACS. Preliminary characterization demonstrated that GFP+OCPs and their derivatives express *Prrx1, Sox9* and mesenchymal cell surface markers SCA1, STRO1 and CD44, although SHP2 deficiency increased the expression of SCA1 and STRO1 (Fig. S3d). 100 ng of total RNA from each was analyzed using Affymetrix GeneChip Mouse Genome 2.0 Arrays. Transcripts that increased or decreased in abundance by >2-fold between SHP2_Prrx1_CTR and SHP2_Prrx1_KO mice, and had a *P* value <0.05 were considered to reflect significant alteration of expression. Of 953 transcripts with differential abundance in OCPs from SHP2_Prrx1_KO;R26^mTmG^ and SHP2_Prrx1_CTR;R26^mTmG^ mice, 397 increased, and 556 decreased. Genes encoding cytoplasmic proteins (12% increased; 20% decreased), ECM proteins (7% increased; 9% decreased), nuclear proteins and transcription factors (8% increased; 7% decreased), plasma membrane proteins (8% increased; 13% decreased) were particularly affected (Fig. 3a).

Chondrocytic and osteoblastogenic genes with altered abundance are shown in Fig. 3b. Among those transcripts that increased in abundance with SHP2 knockout, the greatest changes were in genes previously known to be critical to chondrogenesis and cartilage homeostasis, such as *Acan, Col2a1, Col9a1, Col9a3, Col11a1, Comp, Matn1, Matn3, and Matn4*. Most of these genes harbor binding sites for SOX9 (Fig. 3b asterisks; S4).^48,49^ Genes known to be involved in osteoblastogenesis, such as *Mmp13* and *Ptch1*, however, were downregulated. The differential gene expression observations were confirmed by qRT-PCR analyses, which verified the upregulation of several chondrocytic gene transcripts, such as *Acan, Col2a1, Col10a1, Sox9, Ihh, Alpl,* and *PthrP* in OCPs and their derivatives from SHP2_Prrx1_KO;R26^mTmG^ mice, compared to SHP2_Prrx1_CTR;R26^mTmG^ controls. By contrast, expression of the adipogenic marker *Ppar-r* and the osteogenic marker *Col1α1* were comparable in the cells from these mice (Fig. 3c). There are two potential interpretations for these findings: increased expression of chondrocytic transcripts in SHP2-deficient committed chondrocytes, and/or increased numbers of cells committed to the chondroid lineage. We hope to differentiate between these two possibilities in future experiments by employing single cell RNA sequencing methods.

To gain insight into the mechanism through which SHP2 regulates skeletogenesis, we used Ingenuity® Pathway Analysis (IPA®, Qiagen) to predict the pathways affected in the differential gene expression profiles from SHP2_Prrx1_CTR;R26^mTmG^ and SHP2_Prrx1_KO;R26^mTmG^ OCPs. The top 20 predicted signaling pathways are listed in Fig. 3d, and included the protein kinase A (PKA), RAS/ERK, WNT/β-CATENIN, integrin, p70S6 kinase, PI3 kinase/AKT, PTEN, IGF1, and mTOR. Overall, pathways involving cell development processes and post-translational modification were the most frequently identified (Fig. S4p3). Many of these have been previously associated with skeletal development.

### Enhanced chondrogenesis is associated with increased SOX9 abundance in SHP2_Prrx1_KO mice

**Fig. 4:**
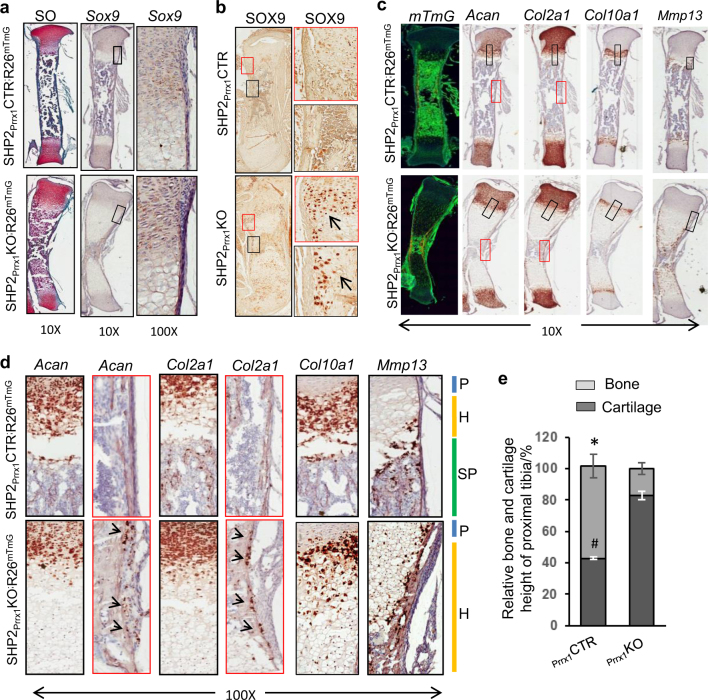
*Ptpn11* deletion in OCPs increases SOX9 abundance and cartilage-associated gene expression. **a** Tibiae sections from 1.5-day-old mice, stained with Safranin O/fast green (left) or hybridized with a murine probe against *Sox9* (middle). Note the persistence of chondroid-appearing cells and the abundance of *Sox9* in cells within the dense soft tissue layer adjacent to the unmineralized femoral anlagen, periosteal area and proliferating chondrocytes of growth plates in SHP2_Prrx1_KO mice, compared within SHP2_Prrx1_CTR mice. Enlarged view of the corresponding black-boxed areas is presented on the right. **b** Images of femoral growth plate and periosteum sections of 7-day-old mice, immunostained with antibodies against SOX9. Note the elevated abundance of SOX9 in cells within periosteal area (black boxes) and proliferating and hypertrophic chondrocytes (red boxes) in growth plates in SHP2_Prrx1_KO mice, compared with SHP2_Prrx1_CTR mice. **c** Representative cryotome sections of tibias from 1.5-day-old SHP2_Prrx1_CTR;R26^mTmG^ and SHP2_Prrx1_KO;R26^mTmG^ mice, hybridized to probes against murine *Acan, Col2α1, Col10α1*, and *Mmp13*. Note that there is an increase in the abundance of transcripts and more cells expressing *Acan, Col2α1*, and *Col10α1*, and *Mmp13* in the periosteal cells *(arrows)*. MMP13 abundance is elevated only in the periosteal areas of SHP2_Prrx1_KO mice. Enlarged views of corresponding color-boxed areas are presented in figure panel (**d**). Layers of proliferating and hypertrophic chondrocytes and metaphysis are indicated with blue, yellow and green bars respectively on the right. Magnification is listed below (*n* = 3). **e** Bar graphs demonstrate the relative endochondral bone (light gray) and epiphyseal cartilage (dark gray) heights (%) of the tibiae from P0.5 neonates; (*n* = 3, *^#^*P* < 0.01, Student’s *t* test).

Given the elevated abundance of several chondrocytic genes in OCPs and their derivatives from SHP2_Prrx1_KO;R26^mTmG^ mice, we next evaluated which cell population in the epiphyseal cartilage of SHP2_Prrx1_KO;R26^mTmG^ mice was affected by SHP2 deficiency in vivo. Entire tibiae were collected from 1.5-day-old SHP2_Prrx1_CTR;R26^mTmG^ and SHP2_Prrx1_KO;R26^mTmG^ mice. After fixation, frozen sections were subjected to in situ hybridizations to evaluate the mRNA abundance of *Sox9*, *Acan*, collagen types II (*Col2α1*) and X (*Col10α1*), and *Mmp13*, and by immunostaining to visualize SOX9 expression. Compared with SHP2_Prrx1_CTR;R26^mTmG^ mice, *Sox9* transcript abundance was only slightly increased in the perichondrium and growth plate cartilage of SHP2_Prrx1_KO;R26^mTmG^ mice (Fig. 4a). However, immunostaining revealed significantly increased SOX9 levels in both of these cell types suggesting that the SOX9 protein persisted with SHP2 knockout (Fig. 4b, arrows), with corresponding increases in the abundance of *Acan, Col2α1*, and *Col10α1* mRNAs in the growth plate cartilage and periosteal cells of SHP2_Prrx1_KO;R26^mTmG^ mice, compared with SHP2_Prrx1_CTR;R26^mTmG^ controls (Fig. 4c, d; S5). No apparent difference in *Mmp13* mRNA or MMP13 protein was detected in the growth plate cartilage of SHP2_Prrx1_CTR;R26^mTmG^ and SHP2_Prrx1_KO;R26^mTmG^ mice, whereas their levels were increased in the perichondral areas of SHP2_Prrx1_KO;R26^mTmG^ mice (Fig. 4c, d arrows; S5). Consistent with these findings, cartilage and ossified endochondral bone comprised 82.6% and 17.4% of tibia length, respectively, in SHP2_Prrx1_KO;R26^mTmG^ mice, compared with 42.8% and 57.2% in SHP2_Prrx1_CTR;R26^mTmG^ mice (Fig. 4e). The increased abundance of SOX9, *Acan*, *Col2α1, Col10a1*, and *Mmp13* in the epiphysis and perichondrial areas of SHP2_Prrx1_KO;R26^mTmG^ mice provides further support for our interpretation that SHP2 deficiency in OCPs causes enhanced chondrocytic differentiation mediated by SOX9.

### SHP2 regulates SOX9 abundance by modifying its phosphorylation and SUMOylation

With strong evidence that the fate of PRRX1-expressing OCPs is regulated by SHP2 via effects on SOX9 levels, we sought to explore the underlying mechanism. SOX9 abundance is tightly controlled by multiple signaling pathways, at both the transcriptional and post-translational levels.^50^ We focused on phosphorylation and SUMOylation, for several reasons. First, SOX9 is expressed in various tissue stem cells, including OCPs,^51,52^ and plays a crucial role in fate determination, survival and proliferation.^16,53,54^ Importantly, it has been reported that SOX9 is stabilized by phosphorylation and SUMOylation via the PKA signaling pathway.^24,25^ Second, pathway analysis of our array data predicted that PKA signaling is substantially affected by SHP2 deficiency in OCPs and their derivatives (Fig. 3d). And third, we found increased SOX9 abundance, but not *Sox9* mRNA in SHP2_Prrx1_KO OCPs, which strongly suggested post-translational control.

**Fig. 5:**
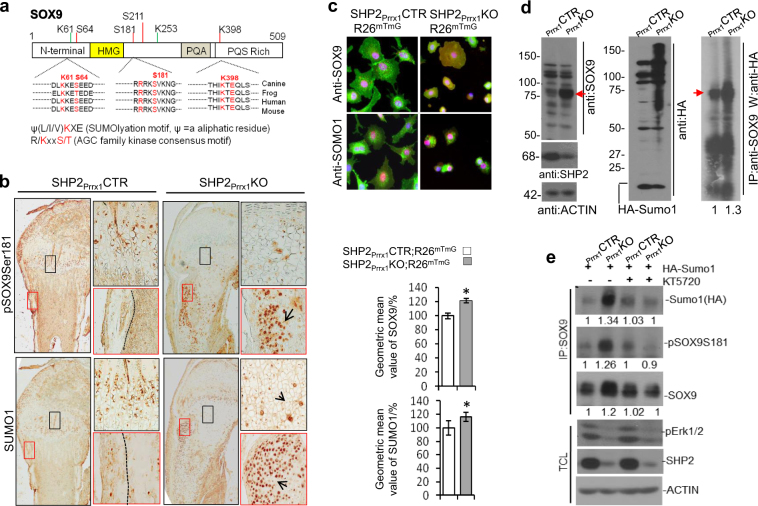
SHP2 regulates SOX9 abundance by modifying its phosphorylation and SUMOylation. **a** Diagrams showing the putative AGC family kinase phosphorylation motifs and SUMOylation sites on murine SOX9. **b** Images of immunostained tibial growth plate and periosteal sections, demonstrating enhanced protein SUMOylation and phosphorylation of SOX9 in 7-day-old SHP2_Prrx1_KO mice, compared with controls. Enlarged view of corresponding boxed areas in the periosteal regions and tibia growth plates are shown on the right (*n* = 3 for each genotype). **c** Representative fluorescence microscopic images (top) and bar graphs of geometric mean values of SOX9 and SUMO1 abundance, as determined by flow cytometric analysis (bottom), demonstrating that the abundance of SOX9 (red) and protein SUMOylation (red) were increased in SHP2-deficient OCPs and their derivatives, compared with controls (*n* = 3 for each genotype, *P < 0.05, Student’s *t* test). **d** Western blot analysis of total cell lysates (left and middle) and anti-SOX9 Immunoprecipitates (right) demonstrating the elevated abundance and SUMOylation of endogenous SOX9 (red arrows) in SHP2-deficient (KO) vs. -sufficient (CTR) OCPs that had been transiently transfected with pcDNA3/HA-Sumo1 plasmids. Note the increase of overall protein SUMOylation in SHP2-deficient OCPs. **e** Western blot analysis showing that blocking PKA activation in SHP2-deficient (cKO) chondroprogenitors with the inhibitor KT5720 compromises SOX9 phosphorylation, SUMOylation and its abundance. Images in **d** and **e** are representative of three experiments. Quantitative data relative to the controls are provided beneath each blot. TCL total cell lysate.

There are 2 AGC family kinase consensus motifs and 2 SUMOylation sites in SOX9 (Fig. 5a), but Ser^181^ and Lys^398^ have been reported to be the primary phosphorylation and SUMOylation sites, respectively.^24,25^ Accordingly, we immunostained tibia sections from 7-day-old mice for SUMO1 and pSOX9Ser^181^. We observed increased protein SUMOylation and SOX9 Ser^181^ phosphorylation (Fig. 5b) in the cells within the periosteal areas and growth plate cartilage of SHP2_Prrx1_KO mice, compared with SHP2_Prrx1_CTR controls. We confirmed these findings using FACS sorting for GFP^+^ OCPs and their derivatives from 1–3 day-old mice, and immunostaining with anti-SOX9 and -SUMO1 antibodies, respectively (Fig. 5c, top). The geometric means of the SOX9 and SUMO1 signals were significantly elevated upon *Ptpn11* deletion (Fig. 5c, bottom). We obtained similar results using control and *Ptpn11*-knockdown ATDC5 cells that transiently expressed GFP-tagged SUMO1 and RFP-tagged human SOX9 (Fig. S6a).

The above experiments did not prove that the elevated protein SUMOylation in SHP2-deficient mice in vivo and in OCPs in vitro affected SOX9 abundance. To address this question, HA-tagged SUMO1 was transfected into SHP2-sufficient and deficient chondroprogenitors. Immunoprecipitation and western blot analyses demonstrated that SOX9 was indeed SUMOylated, and its SUMOylation was enhanced in the absence of SHP2. Most importantly, SOX9 protein abundance in SHP2-deficient chondroprogenitors was higher than in SHP2-sufficient cells (Fig. 5d). Given that SOX9 SUMOylation is augmented by phosphorylation on Ser181,^55^ and the fact that the PKA signaling pathway ranked highly in the Ingenuity pathway analysis, it seemed likely that PKA would function downstream of SHP2 to regulate the phosphorylation and SUMOylation of SOX9. To test this hypothesis, groups of SHP2-sufficient and deficient chondroprogenitors were transfected with HA-tagged SUMO1. After 72 h of incubation, half of the transfected cells were exposed to the PKA inhibitor KT5720 for 6 h. Cells were then lysed, and SOX9 was immunoprecipitated and its phosphorylation and SUMOylation were evaluated by immunoblotting. PKA inhibition markedly compromised SOX9 phosphorylation (as judged by SOX9Ser181 phosphorylation) and SUMOylation in SHP2-deficient chondroprogenitors. KT5720 acts as a competitive antagonist of ATP at its binding site on the PKA catalytic subunit. Like other protein kinase inhibitors, KT5720 can have off-target effects and inhibits other protein kinases, such as ERK, when it exceeds its optimal dosage. We monitored ERK inhibition as a readout of PKA off-target effects in this study and found comparable pERK activation in KT5720-treated and untreated OCPs. These data together suggest that PKA activity in SHP2-deficient chondroid cells stabilizes SOX9 and enhances chondrogenesis (Fig. 5e).

### SHP2 regulates lineage commitment of mesenchymal cells by tilting the balance of SOX9 and β-CATENIN expression

**Fig. 6:**
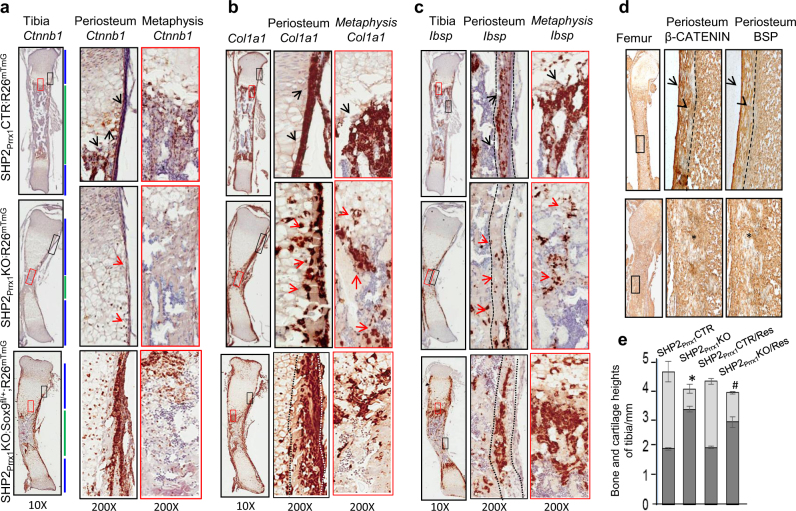
SHP2 deficiency compromises osteogenic differentiation of OCPs and skeletal ossification, and can be rescued by haploinsufficiency of *Sox9*. **a–c** Frozen sections from 1.5-day-old mouse tibiae, hybridized to probes for murine *Ctnnb1* (**a**), *Col1α1* (**b**), and *Ibsp* (**c**). Enlarged views of the corresponding color-boxed areas are presented on the right. Note the reduced abundance of *Ctnnb1, Col1α1*, and *Ibsp* in cells within the periosteum and spongy bone in SHP2_Prrx1_KO mice (middle row), compared with SHP2_Prrx1_CTR mice (top row). These defects and defective ossification were partially rescued by *Sox9* haploinsufficiency in SHP2_Prrx1_KO;Sox9^fl/+^;R26^mTmG^ mice (bottom row). **d** Images of femurs from 7-day-old SHP2_Prrx1_CTR and SHP2_Prrx1_KO mice, immunostained with antibodies against β-CATENIN and bone sialoprotein (BSP). Note the reduction of β-CATENIN and BSP abundance in periosteal areas (_*_) of SHP2_Prrx1_KO mice, compared with SHP2_Prrx1_CTR controls (arrows). Arrowheads indicate calcified cortical bone in SHP2_Prrx1_CTR mice (**a–d**, *n* = 3). **e** Bar graphs demonstrate the heights of epiphyseal cartilage (dark gray) and endochondral bone (light gray) of tibia from P0.5 neonates. Note that removal of one allele of *Sox9* significantly increased the length of endochondral bone in SHP2_OCP_KO^Res^ mice, compared to controls (*n* = 3, *^#^*P* < 0.05, 2-way ANOVA).

Having established a role for SHP2 in the regulation of SOX9 and chondrogenesis, we next examined how SHP2 deficiency in PRRX1-expressing OCPs affects osteogenesis in SHP2_Prrx1_KO mice. β-CATENIN activity is crucial for the differentiation of OCPs into bone cells, and mice lacking β-CATENIN in OCPs fail to form osteoblasts and calvarial bone.^56,57^ Furthermore, β-CATENIN protein abundance has been shown to be antagonistically regulated by SOX9.^13^ Given our finding of defective endochondral and intramembranous bone formation and elevated SOX9 abundance in SHP2_Prrx1_KO mice, we examined the effect of *Ptpn11* deletion on β-CATENIN and *Ctnnb1* abundance in the periosteal and bone cells of SHP2_Prrx1_CTR and SHP2_Prrx1_KO mice. In situ hybridization revealed reduced *Ctnnb1* abundance in cells within the periosteal and metaphyseal regions of SHP2_Prrx1_KO mice, compared with SHP2_Prrx1_CTR controls (Fig. 6a). Consistent with these findings, the expression of other osteogenic genes, such as *Col1α1* and *Ibsp*, also was decreased (Fig. 6b, c). These observations were further supported by the reduction of β-CATENIN and bone sialoprotein (BSP) abundance in the corresponding cells in SHP2_Prrx1_KO mice, compared to SHP2_Prrx1_CTR (Fig. 6d). By contrast, *Sox9, Col2α1,* and *Acan* were expressed at high levels in SHP2-deficient OCPs and their derivatives (Fig. 4a–d).

To provide additional evidence that elevated SOX9 directly influences the skeletal phenotype in SHP2_Prrx1_KO mice, we used a genetic rescue approach. A single *Sox9* floxed allele was bred to SHP2_Prrx1_CTR;R26^mTmG^ and SHP2_Prrx1_KO;R26^mTmG^ mice. After serial breeding we were able to generate lines of SHP2_Prrx1_CTR;R26^mTmG^ and SHP2_Prrx1_KO;R26^mTmG^ mice that were haploinsufficient for *Sox9* in PRRX1-expressing cells (SHP2_Prrx1_CTR;Sox9^fl/+^;R26^mTmG^ and SHP2_Prrx1_KO;Sox9^fl/+^;R26^mTmG^). Compared with SHP2_Prrx1_CTR;Sox9^+/+^;R26^mTmG^ controls, SHP2_Prrx1_CTR;Sox9^fl/+^;R26^mTmG^ mice had no discernible phenotype. By contrast, haploinsufficiency of SOX9 in PRRX1-expressing cells in SHP2_Prrx1_KO mice (SHP2_Prrx1_KO;Sox9^fl/+^;R26^mTmG^) markedly restored endochondral ossification compared with SHP2_Prrx1_KO;Sox9^+/+^;R26^mTmG^ mice, which manifested as an increase of the primary ossification center and expression of *Ctnnb1, Col1α1* and *Ibsp* in periosteum and spongiosa bone (Fig. 6a–c). Accordingly, the length of the epiphyseal cartilage and primary ossification center was reduced and increased, respectively, in SHP2_Prrx1_KO;Sox9^fl/+^;R26^mTmG^ mice (Fig. 6e, S6b). Collectively, these data suggest that elevated SOX9 in PRRX1-expressing cells of SHP2_Prrx1_KO mice plays a central role in chondroid cell development and abnormal endochondral ossification.

### Mosaic SHP2 deficiency in PRRX1-expressing OCPs causes exostoses and enchondromas

**Fig. 7:**
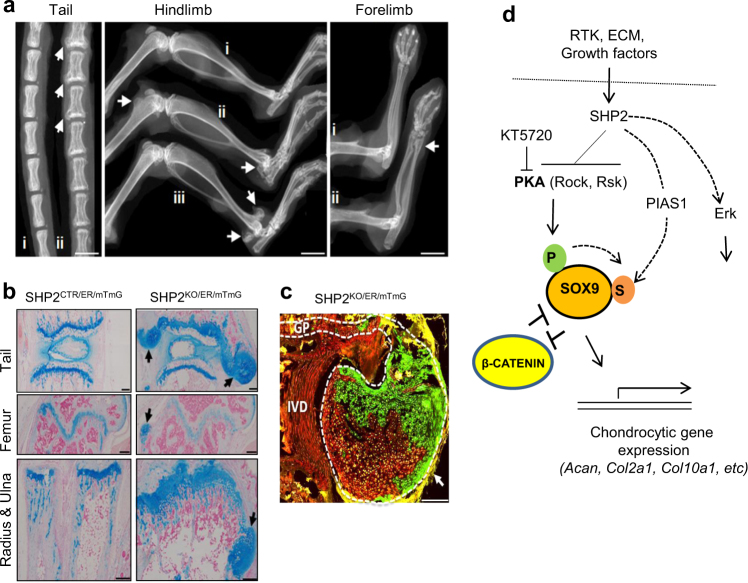
Mosaic *Ptpn11* deletion at E13.5 in *Prrx1-CreERt2*-expressing OCPs leads to osteochondromas and enchondromas. **a** High resolution plain radiographs showing the outgrowth of cartilaginous lesions (arrows) on the tail vertebrae, distal femur, distal tibia, radius and ulna (*n* = 14). i: SHP2^CTR/ER/mTmG^; ii, iii:SHP2^LOH/ER/mTmG^. **b** Images of tail vertebrae, distal femur, and ulna sections, stained with Alcian blue and nuclear fast red, demonstrating cartilaginous lesions (arrows) in SHP2^KO/ER/mTmG^ mice, compared with SHP2^CTR/ER/mTmG^ mice. Note that there is no clear separation between the growth plates of the radius and ulna in SHP2^LOH/ER/mTmG^ mice. **c** Merged fluorescent microscopic images showing cells in which the *Rosa26*^*mTmG*^ reporter allele has (green fluorescence) or has not (red fluorescence) been recombined by Cre recombinase in a SHP2^LOH/ER/mTmG^ mouse. A large cartilage outgrowth (arrow) adjacent to the intervertebral disc (IVD) and tail vertebral growth plate (GP) is shown. The GP and cartilage outgrowth are outlined (white dashed line). Note that green and red chondrocytes are observed in the outgrowth. Scale bars in **a** represent 4 mm; Scale bars in **b** and **c** represent 250 μm (For the studies in **b** and **c**, *n* = 3 for each genotype). **d** Proposed model of how SHP2 regulates chondrogenesis and endochondral bone formation by altering the expression of the transcription factors SOX9 and β-CATENIN. P, phosphorylation; S, SUMOylation.

SHP2 loss-of-function (LOF) mutations in humans are found in families segregating metachondromatosis, where a somatic second-hit mutation is postulated to be responsible for the development of cartilage lesions. In mice, homozygous SHP2 LOF mutations in COL2α1-expressing chondroid cells and the cathepsin K-expressing (CTSK^+^) groove of Ranvier cells produce enchondromas (cartilaginous masses formed inside bone) and exostoses (outgrowth of cartilage capped masses on bone surfaces) similar to those found in metachondromatosis patients.^35,37,39,58^ In patients, the cell-type(s) in which a second hit mutation causes cartilage lesions is unknown. It is possible the cells involved are osteochondral progenitors. Accordingly, we investigated whether second hits (loss-of-heterozygosity, LOH) in murine OCPs might cause cartilaginous lesions by breeding mice with *Ptpn11* floxed, null (Δk11), and *R26*^*mTmG*^ reporter allele to *Tg(Prrx1-CreERt2)* mice that express a tamoxifen-inducible Cre under the control of the *Prrx1* promoter.^41^ Pregnant dams were administered one low dose of TM at E13.5 (50 mg·kg^-1^), and *Tg(Prrx1-CreERt2;Ptpn11*^*fl/Δk11*^*;R26*^*mTmG*^*)* (SHP2^LOH/ER/mTmG^) and *Tg(Prrx1-CreERt2;Ptpn11*^*fl/+*^*;R26*^*mTmG*^*)* (SHP2^CTR/ER/mTmG^) offspring were identified postnatally and evaluated for the development of skeletal disease. SHP2^CTR/ER/mTmG^ and SHP2^LOH/ER/mTmG^ mice were born at the expected Mendelian ratios and had normal gross appearance within the first 8 weeks after birth. By 11 weeks of age, however, 86% (12/14) of SHP2^LOH/ER/mTmG^ mice had noticeable exostoses, mostly in the vertebrae and at the end of tubular bones (Fig. 7a). In addition, the growth plates of the radius and ulna were merged in SHP2^LOH/ER/mTmG^ mice (Fig. 7b). By contrast, SHP2^CTR/ER/mTmG^ mice appeared phenotypically normal. The Rosa26^mTmG^ reporter study revealed that exostotic lesions in SHP2^LOH/ER/mTmG^ mice were comprised of both green (recombined) and red (non-recombined) chondroid cells (Fig. 7c), suggesting that wild-type and SHP2-deficient chondroid cells participate in the growth of exostoses. Interestingly, SHP2^LOH/ER/mTmG^ mice that received TM injection at postnatal week 2 had no apparent enchondromas or exostoses detectable by micro-CT when they were killed at post-natal week 12 (Fig. S7). These data demonstrate that SHP2 has a critical time-dependent role in modulating the proliferation and chondrocytic differentiation of OCPs and that aberrant SHP2 signaling in OCPs can lead to neoplastic cell growth and cartilage tumor formation at certain developmental stages.

## Discussion

SHP2 is a ubiquitously expressed cytoplasmic protein-tyrosine phosphatase that has cellular context-specific roles in regulating the viability, proliferation, and differentiation of various types of cells and tissues.^31,32^ In this study, we found that SHP2 influences skeletal phenotype through SOX9. SHP2 was specifically ablated in PRRX1-expressing osteochondroprogentors (OCPs), a subpopulation of mesenchymal cells with the ability to form appendicular bone and cartilage. Mice lacking SHP2 in PRRX1-expressing OCPs displayed a broad range of skeletal abnormalities, including defective ossification of the skull and long bones, small and deformed concave- or convex-shaped ribcages, and short and deformed limbs and joints affecting the hip, knee, ankle and phalanges. Interestingly, a few of the surviving mice developed localized hypertrichosis on the forelimbs and hind limbs.^59^ Lineage tracing suggested that the skeletal phenotypes of SHP2_Prrx1_KO mice are OCP-autonomous. Gene arrays and pathway analyses revealed differential expression of 953 genes in SHP2-deficient OCPs, and more than 20 primary signaling pathways were affected substantially by SHP2 deficiency. Among significantly upregulated were genes known to be critical for chondrogenesis, such as *Col2a1*, *Col10a1*, *Ihh, PthrP*, *Acan*, and *Sox9*, while genes involved in osteoblastogenesis, such as *Sp7* and *Ctnnb1*, decreased in abundance. The chondrocytic master transcription factor SOX9 was elevated in cells within the periosteal areas and “cartilage islands” of the bone marrow compartment of SHP2_Prrx1_KO mice. Most importantly, haploinsufficiency of SOX9 in the PRRX1-expressing cells partially rescued the defective endochondral ossification and epiphyseal cartilage development in SHP2_Prrx1_KO mice.

Mechanistically, we found that SHP2 regulates SOX9 abundance in OCPs and chondrocytic cells by modulating the phosphorylation and SUMOylation of SOX9. Our data are consistent with previous reports that SOX9 contains target sites for the AGC family serine/threonine kinases PKA, RSK2, and ROCK.^55^ Moreover, SOX9 interacts physically with PKA, and phosphorylation of SOX9 at Ser64 and Ser181 by PKA modulates its transcriptional activity.^25,60^ Using conditional knockout mice and PRRX1-expressing chondroprogenitor cell lines, we found that SOX9 protein abundance was elevated when SHP2 was deficient. PKA inhibition with KT5720 lowered the amount of SOX9 in control and SHP2-deficient cells in vitro, suggesting that PKA regulation is downstream of SHP2. This observation was also supported by the localized hypertrichosis in some of the surviving SHP2_Prrx1_KO mice. It has been reported that increased abundance of SOX9, due to the deficiency of *Trps1* in the hair follicle, accounts for the growth of long hairs.^44^ Given that Prrx1-Cre activity has not been observed in limb ectoderm,^40^ the hypertrichotic phenotype in *Tg(Ptpn11*^*fl/fl*^*;Prrx1-Cre)* mice might be caused by miscommunication between PRRX1+ SHP2-deficient mesoderm cells and ectodermal cells within the hair follicle. Further studies will be required to delineate the specific cellular and molecular mechanisms behind these intriguing observations.

SUMOylation is a multistep enzymatic process that leads to the covalent attachment of a small ubiquitin-like modifier (SUMO) protein to a lysine residue of a target protein. This modification can regulate the activity, stability, subcellular localization, and the repertoire of interactions of targeted proteins.^61,62^ Two putative SUMOylation sites have been identified in murine SOX9—K61 and K396. Of these, K396 has been shown to be the primary SUMOylation site.^63^ We demonstrated that SOX9 SUMOylation is increased in SHP2-deficient OCPs and their derivatives, and that SUMOylation is associated with increased expression of *Col2a1*, *Col10a1, Aggrecan, Ihh*, and *Pthrp*. SUMOylation of SOX9 on K396 affects its stability and activity, leading to an increase in both the abundance and transcriptional activity of SOX9.^24,64,65^ However, it has also been reported that SOX9 SUMOylation represses its transcriptional activity in 293 T cells in vitro.^63^ The reason for this discrepancy remains unclear, but it might reflect the two distinct cellular contexts and the reporter systems used.

Increasing evidence indicates that SOX9 levels and transcriptional activity are also regulated by the ubiquitin-proteasome-mediated degradation pathway. Inhibition of the 26 S proteasome by MG132 increases SOX9 activity. In addition, mutation of K398, the primary site for ubiquitination and SUMOylation of SOX9, increased SOX9 protein stability and transcriptional activity.^66^ Taken together, these data suggest a model where SUMO molecules compete with ubiquitin molecules to bind to K396. The balance between SOX ubiquitination and SUMOylation likely controls the chondrogenic processes.

SHP2 loss-of-function (LOF) mutations in humans causes the autosomal dominant, incompletely penetrant, cartilage tumor syndrome “metachondromatosis”. However, the cell-type(s) in which second hit *Ptpn11* mutation causes cartilage lesions remains unclear. We created a *Ptpn11* LOH mouse model in PRRX1-expressing cells, and found that SHP2 deficiency in PRRX1+ OCPs was involved in the pathogenesis of cartilage lesions, indicating that SHP2 functions as a tumor suppressor in cartilage and is required in OCPs for cartilage development and homeostasis.

In summary, our data support a model in which SHP2 deficiency in OCPs leads to an increase in SOX9, although we do not exclude the possibility that other pathways also are involved. This increase in SOX9 either directs OCP cells towards the chondrocytic and away from the osteogenic lineage or delays their commitment to the osteogenic lineage (Fig. 7d). As a consequence of SHP2 deficiency, abnormal cartilage growths develop at sites where mineralized bone would normally form, including the periosteum and primary spongiosum. Our study suggests that other signaling pathway(s) in addition to the SHP2/PKA/SOX9 might also regulates the fate decision of OCPs. The ability to manipulate cell fate choice by modulating SHP2 activity suggests a new strategy to therapeutically manipulate chondrogenesis in patients with a variety of cartilage-related disorders, ranging from tumors to degenerative diseases such as osteoarthritis. Given the “double-edged sword” effects of SHP2 deficiency on chondrogenesis and osteogenesis, caution also must be taken on the skeletal system with the use of SHP2 inhibition^67^ to treat neoplastic diseases.

## Materials and methods

### Transgenic mice

*Sox9* floxed (*Sox9*^*fl*^),^68^ *Ptpn11* floxed (*Ptpn11*^*fl*^) and null alleles (Δk11)^39^ were described previously. The *Tg(Rosa26*^*mTmG*^*)*,^69^ *Tg(Rosa26*^*ZsG*^*)*,^70^ *Tg*(*Prrx1-Cre)*^40^, and *Tg(Prrx1-CreER)*^41^ mice also were reported previously. PCR genotyping conditions for the *Ptpn11* floxed allele, null allele, *Rosa26*^*mTmG*^ and *Rosa26*^*ZsG*^ reporter alleles, and Cre transgenes are described in the original publications and are available upon request. To delete *Ptpn11* in OCPs that express the paired related homeobox-1 protein (PRRX1), mice bearing a *Ptpn11* floxed allele were interbred to *Tg*(*Prrx1-Cre)* or *Tg(Prrx1-CreER)* mice to generate offspring with the following genotypes and nomenclature: *Tg(Prrx1-Cre;Ptpn11*^*fl/+*^*)* (SHP2_Prrx1_CTR), *Tg(Prrx1-Cre;Ptpn11*^*fl/fl*^*)* (SHP2_Prrx1_KO), *Tg(Prrx1-CreERt2;Ptpn11*^*fl/+*^*)* (SHP2_Prrx1_CTR/ER), and *Tg(Prrx1-CreERt2;Ptpn11*^*fl/Δk11*^*)* (SHP2_Prrx1_KO/ER), respectively. To induce haploinsufficiency of *Sox9* in the PRRX1-expressing cells of SHP2_Prrx1_CTR and SHP2_Prrx1_KO mice, a *Sox9*^*fl*^ allele was bred to SHP2_Prrx1_CTR and SHP2_Prrx1_KO mice after serial breeding. To trace PRRX1-expressing cells that lack SHP2 in vivo, SHP2_Prrx1_CTR, SHP2_Prrx1_KO, SHP2_Prrx1_CTR/ER and SHP2_Prrx1_KO/ER mice were also bred with *Tg(Rosa26*^*mTmG*^*)* or *Tg(Rosa26*^*ZsG*^*)* reporter alleles. To induce *Tg(Prrx1-CreER)* activity, 4-OH tamoxifen (TM; Sigma, MO) was dissolved in DMSO-ethanol-corn oil mixture (4:6:90) at a concentration of 10 mg·mL^-1^ and injected intraperitoneally (1 mg per mouse each dose).^71,72^ Control and SHP2 mutant animals were sacrificed at the indicated time points and used for X-ray, histological, biochemical and biological analyses. All transgenic mice were maintained on C57BL/6 background and studied in accordance with the Institutional Animal Care and Use Committee approved protocols.

### Cells and DNA constructs

Primary PRRX1+OCPs and their derivatives were isolated from 1 to 3-day-old pups. To maximize the yield of GFP+OCPs and their derivatives, the epiphyseal portions of knee joints (including distal femurs and proximal tibia) were collected and incubated with trypsin-EDTA (0.25%, Invitrogen) at 37 °C for 60 min. After washing with PBS twice, tissues were further incubated with hyaluronidase (2 mg·mL^-1^; Sigma) for 2 h and hyaluronidase/collagenase D mixture (1 mg·mL^-1^, Roche) for 4 h in DMEM at 37 °C. Undigested bony tissue was discarded by filtration. OCPs and their derivatives were then collected by centrifugation and cultured in DMEM/F12 medium (1:1) (Invitrogen), supplemented with 10% FBS, 1% ampicillin and streptomycin (Invitrogen). After 2–3 passages, GFP+OCP and derivatives were enriched by FACS and used for total RNA isolation. SHP2-sufficient and deficient chondroprogenitor cell lines were established by immortalizing PRRX1-expressing progenitors from 1 to 2-day-old SHP2_Prrx1_CTR;R26^ZsG^ and SHP2_Prrx1_KO;R26^ZsG^ mice with SV40 large T antigen and cultured in DMEM/F12 media supplemented with 10% FBS and 1% penicillin/streptomycin. Plasmids pcDNA3 HA-SUMO1, pEGFP-C1/SUMO1, and pRFP-hSox9 were published^73–75^ and obtained from Dr. Riko Nishimura (Osaka University) and Dr. Akihiro Ito (RIKEN) in Japan, respectively.

### Antibodies and reagents

Polyclonal and monoclonal antibodies (PcAb and McAb) were purchased from commercial sources: PcAb against phospho(p)-ERK1/2, ERK1/2, and McAb against HA were from Cell Signaling Inc.; McAb against murine COL2α1 was from Thermo Scientific; PcAb against MMP13, SOX9 and COL10α1 were from Abcam;. PcAb against SUMO1, IHH, and SHP2 were purchased from Invitrogen, Epitomics and UBI, respectively. PcAb against β-CATENIN were purchased from Cell Signaling (MA). Anti-BrdU antibodies and BrdU staining kit were obtained from BD pharmingen. PE-conjugated, affinity purified PcAb against SOX9 were purchased from Bioss. The PKA inhibitor KT5720 was purchased from Santa Cruz. PE-conjugated anti-Rabbit IgG was purchased from Cell Signaling. Alcian blue and Alizarin Red S staining solutions were purchased from Poly Scientific.

### Histological analysis

To examine the effect of SHP2 deficiency on gross skeletal development, mice were eviscerated after euthanasia and fixed in 4% paraformaldehyde (PFA) overnight at 4°C. Fixed skeletons were stained with Alcian Blue and Alizarin Red to visualize cartilage and calcified bones. Femurs and tibia from SHP2_Prrx1_CTR and SHP2_Prrx1_KO mice were decalcified, sectioned and stained with hematoxylin and eosin (H&E), Alcian blue and Safranin O/fast green for morphologic analysis, and with antibodies against chondrogenic and osteogenic markers to evaluate cartilage and bone development. To trace the fate of PRRX1-expressing OCPs in mice, femurs and tibiae were collected from 1- to 2-day-old SHP2_Prrx1_CTR;R26^mTmG^ and SHP2_Prrx1_KO;R26^mTmG^ mice and frozen section were used to visualize green fluorescent protein (GFP)-positive cells microscopically. DAPI was used to counterstain the nucleus. All fluorescent and phase contrast images were obtained using a Nikon digital fluorescence microscope and an Aperio slide scanner (Vista, CA). Immunostaining was carried out using Vectorstain ImmPACT/DAB kit following the manufacturer’s instructions.

### Gene expression array and quantitative RT-PCR analyses

Total RNA was extracted using RNeasy kit (Qiagen) from short-term-expanded and FACS-enriched primary OCPs and their derivatives from 3-day-old SHP2_Prrx1_CTR;R26^mTmG^ and SHP2_Prrx1_KO;R26^mTmG^ mice, and analyzed for integrity by using an Agilent 2100 Bioanalyzer. For differential gene expression analysis, three RNA samples per mouse line were amplified using Invitrogen WT Expression kit and hybridized to Affymetrix Mouse Gene ST 2.0 arrays. Ingenuity Pathway Analysis (IPA) Software from Qiagen was used for pathway analysis.

To validate the expression of differentially regulated genes on arrays, qRT-PCR was performed with RT^2^SYBR®Green qRT-PCR kit on a Bio-Rad CFX machine using cDNA that was synthesized using 1 µg total RNA with iScript™cDNA Synthesis Kit (Bio-Rad). All samples were normalized to *Gapdh* and gene expression data were presented as fold-increases or -decreases compared with controls. All primer sequences used for this study are available by request.

### In situ hybridization

Femurs and tibiae were collected from post-natal day 1.5 neonates. After fixation in 4% paraformaldehyde overnight, 7 µm cryostat sections were used for in situ hybridization with probes against murine *Sox9, Acan, Col2α1, Col10α1, Col1α1, Ctnnb1, Ibsp*, and *Mmp13*. Hybridization and detection of hybridization signals were achieved using RNAscope HD-Brown kit per the manufacture’s instruction (Advanced Cell Diagnostics).

### Flow cytometry analysis

Primary OCPs and their derivatives from SHP2_Prrx1_CTR;R26^mTmG^ and SHP2_Prrx1_KO;R26^mTmG^ mice were either analyzed or purified by FACS for GFP+ cells. Purified GFP+ OCPs and their derivatives were fixed, permeabilized,^76^ and stained with PE-conjugated anti-SOX9 or SUMO1 antibodies. All samples were subjected to FACS using a BD LSRII flow cytometer (San Jose, CA) and analyzed using the FlowJo software.

### Immunoprecipitation and western blot analysis

Cells were lysed in modified NP-40 lysis buffer [0.5% NP40, 150 mmol·L^-1^ NaCl, 1 mmol·L^-1^ EDTA, 50 mmol·L^-1^ Tris (pH 7.4)], supplemented with a protease inhibitor cocktail (1 mmol·L^-1^ PMSF, 10 mg·mL^-1^ aprotinin, 0.5 mg·mL^-1^ antipain, and 0.5 mg·mL^-1^ pepstatin). Immunoprecipitations were performed on cleared lysates, as described previously.^77^ For immunoblotting, cell lysates (30–50 µg) were resolved by SDS-PAGE, transferred to PVDF membranes, and incubated with primary antibodies for 2 h or overnight at 4 °C (according to the manufacturer’s instructions), followed by HRP-conjugated secondary antibodies (Bio-Rad).

### Induction of exostotic lesions in SHP2^LOH/ER^;R26^mTmG^ mice

Timed matings were performed with 8 to 12-week-old females caged overnight with males, and vaginal plugs were checked the following morning. Fertilization was assumed to occur at midnight, and the time of plug identification was defined as E0.5. TM was administered to pregnant females at E13.5 (50 mg·kg^-1^) and pups with the genotypes *Tg*(*Prrx1-CreERt2;Ptpn11*^*fl/Δk11*^*;R26*^*mTmG*^*)* (SHP2^LOH/ER/mTmG^) and *Tg(Prrx1-CreERt2;Ptpn11*^*fl/+*^*;R26*^*mTmG*^*)* (SHP2^CTR/ER/mTmG^) were identified by PCR after birth. SHP2^CTR/ER/mTmG^ and SHP2^LOH/ER/mTmG^ mice were sacrificed at post-natal week 11 or 15 for radiographic and histologic analysis, as described earlier. X-ray images were taken immediately after euthanasia using a digital radiography system (MX-20, Faxitron Bioptics, LLC, Tucson, AZ).

### Statistical analysis

Statistical differences between groups were evaluated with Student’s *t* tests or two-way ANOVAs followed by Holm-Sidak post hoc comparisons. *P* values < 0.05 were considered statistically significant. Analyses were performed by using Prism 3.0 (GraphPad, San Diego, CA), SigmaPlot (Systat Software, Inc. San Jose, CA) and Excel (Microsoft Inc., Redmond, WA).

### Material availability

All mouse lines, DNA constructs and cell lines are available upon request.

## Electronic supplementary material

Supplemental Information(DOCX 7647 kb)
