# Supplementary material for: SHP2 regulates skeletal cell fate by modifying SOX9 expression and transcriptional activity
Source: Bone Res. 2018 Apr 6;6:12. doi: 10.1038/s41413-018-0013-z (PMC5886981; doi:10.1038/s41413-018-0013-z)
Supplement: Supplementary file 1 — Supplemental Information(DOCX 7647 kb) [file 41413_2018_13_MOESM1_ESM.docx]

**SUPPLEMENTARY INFORMATION**

**
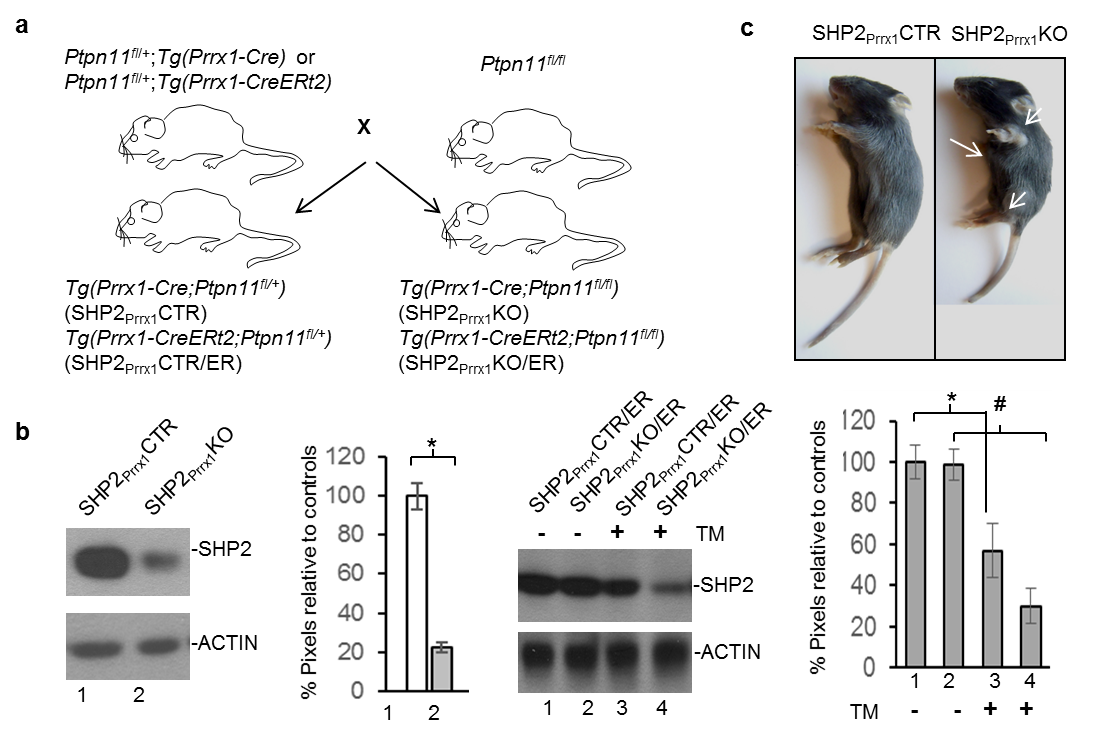
**

**Figure S1.** **Generation of OCP-specific SHP2 deficient mice.** **a.** Diagrams showing the breeding scheme to generate OCP-specific SHP2 deficient mice and littermate controls. **b.** Western blot data demonstrate the abundance of SHP2 in OCPs and their progeny from 2-day-old SHP2_Prrx1_CTR, SHP2_Prrx1_KO, SHP2_Prrx1_CTR/ER and SHP2_Prrx1_KO/ER mice as described in the method section. To induce SHP2 deletion in OCPs from SHP2_Prrx1_CTR/ER and SHP2_Prrx1_KO/ER mice, 4-OH tamoxifen (1µM) was added into the culture medium for 96 hours. Cells were lysed into NP-40 lysis buffer and 30 µg of proteins were loaded for western blot analysis with antibodies against SHP2. ACTIN was used as an internal loading control. Quantitative data are presented as the percentage changes in SHP2 abundance relative to the corresponding controls. All samples were normalized with ACTIN, n=3, *^,#^ *p*<0.05 (Student’s *t* test). TM: 4OH tamoxifen. **c.** Images of the lateral view of a 10-day-old SHP2_Prrx1_KO mouse demonstrating the developmental defects of forelimbs and hindlimbs and a convex-like ribcage; age-matched SHP2_Prrx1_CTR mice served as controls (n=3, each genotype).

**
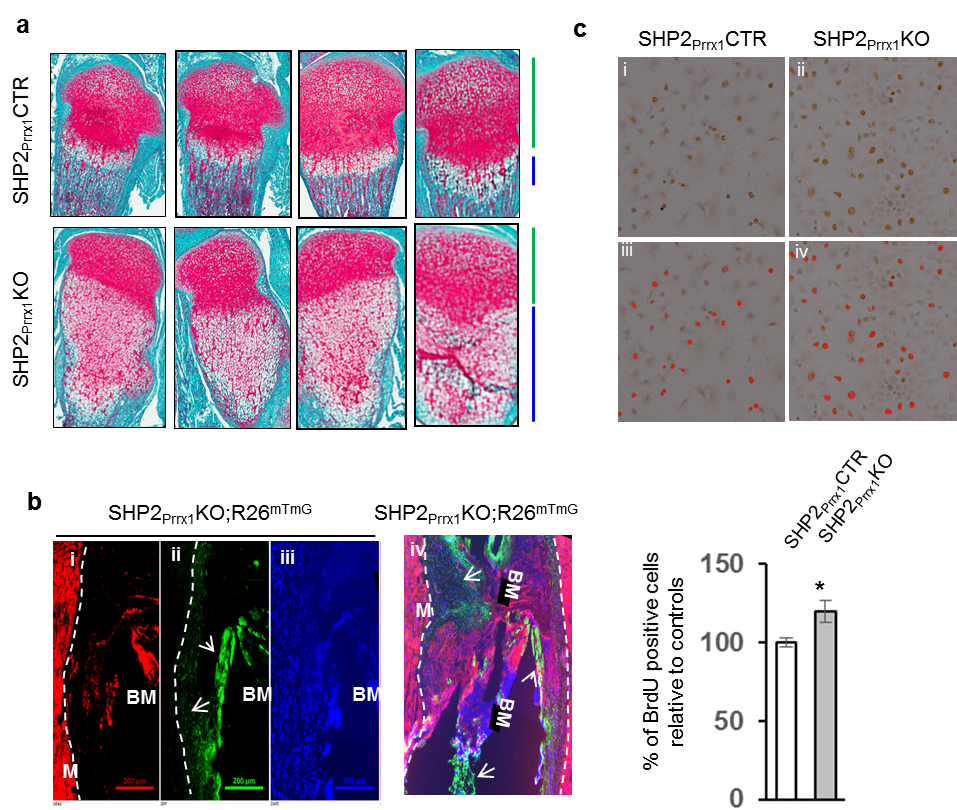
**

**Figure S2.** **a.** Images of safranin O-stained proximal tibia sections from four 3.5-day-old mice demonstrate an increase in cartilage mass and expansion of layers of prehypertrophic and hypertrophic chondrocytes in SHP2_Prrx1_KO mice, compare to SHP2_Prrx1_CTR control. Green and blue bars on the right represent proliferating and hypertrophic chondrocytes respectively. **b.** Fluorescent images of tibia frozen sections demonstrating numerous PRRX1-expressing cells spreading throughout the periosteal areas and bone marrow of SHP2_Prrx1_KO;R26^mTmG^ mice. The bone of SHP2_Prrx1_KO;R26^mTmG^ mice did not mineralize, and the GFP+ OCPs were not restricted to what would have been a thin periosteal soft tissue layer. Dashed lines denote the boundary between muscle and bone. Images of i, ii and iii represent the red, green and blue (DAPI) channel images respectively. **c.** Phase contrast Images (i-iv) of BrdU-labeled OCPs and their progeny isolated from SHP2_Prrx1_CTR;R26^mTmG^ and SHP2_Prrx1_KO;R26^mTmG^ mice. Cells were stained with anti-BrdU antibody and visualized by DAB kit; images of **iii** and **iv** demonstrating BrdU+ cells (red dots) in the images **i** and **ii** identified by NIH ImageJ program . Bar graphs (below) showing the quantitative data of 3 BrdU labeling assays (n=3, **p*<0.05; Student’s *t* test).(**a**-**b**:n=4).

**
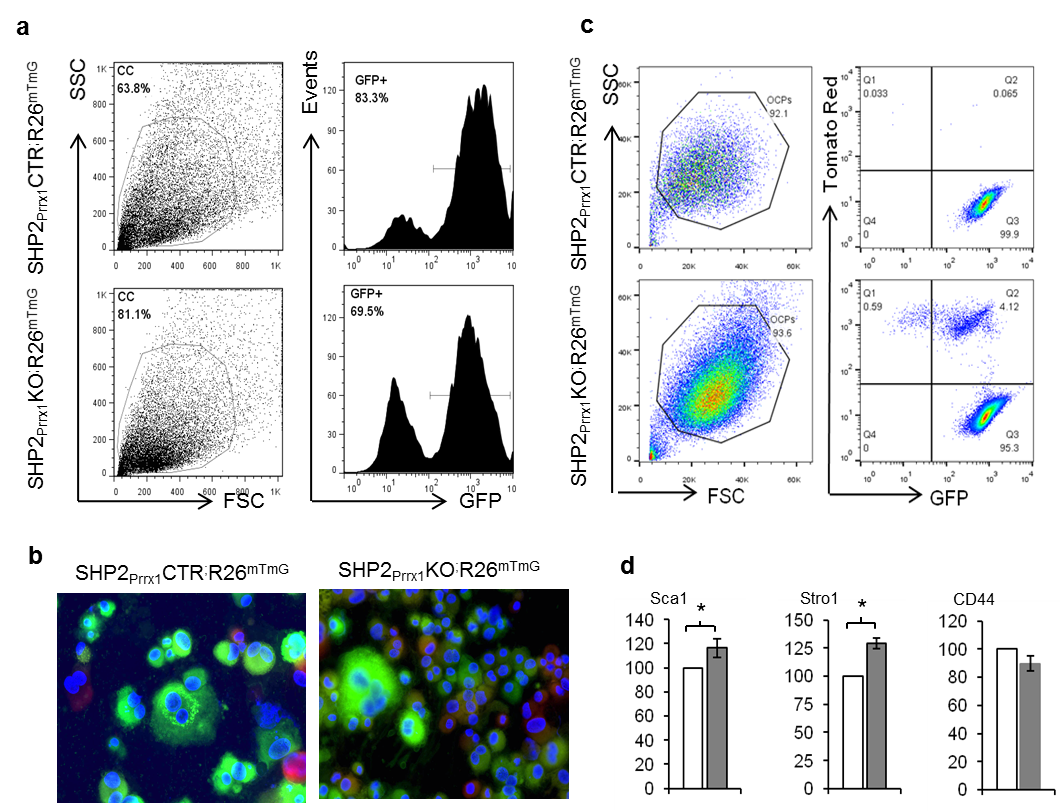
**

**Figure S3.** **a.** Representative flow cytometry data demonstrate that 83.3% and 69.5% of cells were GFP positive in the OCP preparations from SHP2_Prrx1_CTR;R26^mTmG^ and SHP2_Prrx1_KO;R26^mTmG^ mice. **b**. Representative fluorescent microscopic images of OCP prepared from SHP2_Prrx1_CTR;R26^mTmG^ and SHP2_Prrx1_KO;R26^mTmG^ mice. **c**. FACS data demonstrate that majority of OCP were GFP+ post FACS sorting. **d.** FACS analysis demonstrate the relative expression (geometric mean values) of mesenchymal progenitor markers SCA1, STRO1 and CD44 in SHP2_Prrx1_KO;R26^mTmG^ mice, compared with SHP2_Prrx1_CTR;R26^mTmG^ , n=3*, *p<0.05*.

**Figure S4 P1.** A list of selected chondrocytic genes up-regulated in OCPs and their derivatives from SHP2_Prrx1_KO;R26^mTmG^ mice are also down-regulated in the SOX9^-/-^ chondroid cells according to the published literature(see below*). Ingenuity pathway analysis predicting that PKA signaling pathway is mostly affected. *1. C. D. Oh, Y. Lu, S. Liang, Y. Mori-Akiyama, D. Chen, B. de Crombrugghe, H. Yasuda, SOX9 Regulates Multiple Genes in Chondrocytes, Including Genes Encoding ECM Proteins, ECM Modification Enzymes, Receptors, and Transporters. PloS one 9, e107577 (2014). *2. C. D. Oh, S. N. Maity, J. F. Lu, J. Zhang, S. Liang, F. Coustry, B. de Crombrugghe, H. Yasuda, Identification of SOX9 interaction sites in the genome of chondrocytes. PloS one 5, e10113 (2010).

**
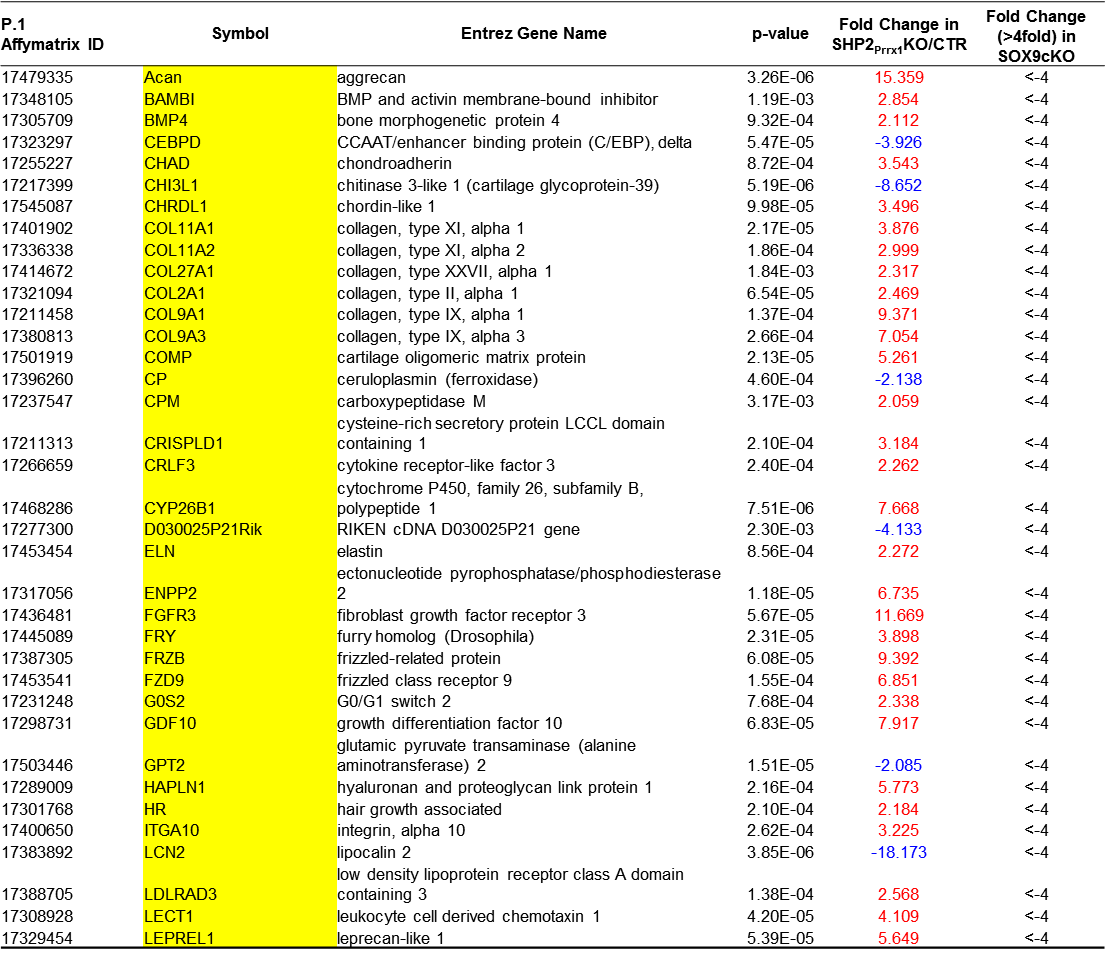
**

**Figure S4 P2.** A list of selected chondrocytic genes up-regulated in OCPs and their derivatives from SHP2_Prrx1_KO;R26^mTmG^ mice are also down-regulated in the SOX9^-/-^ chondroid cells according to the published literature(see below*). Ingenuity pathway analysis predicting that PKA signaling pathway is mostly affected. *1. C. D. Oh, Y. Lu, S. Liang, Y. Mori-Akiyama, D. Chen, B. de Crombrugghe, H. Yasuda, SOX9 Regulates Multiple Genes in Chondrocytes, Including Genes Encoding ECM Proteins, ECM Modification Enzymes, Receptors, and Transporters. PloS one 9, e107577 (2014). *2. C. D. Oh, S. N. Maity, J. F. Lu, J. Zhang, S. Liang, F. Coustry, B. de Crombrugghe, H. Yasuda, Identification of SOX9 interaction sites in the genome of chondrocytes. PloS one 5, e10113 (2010).


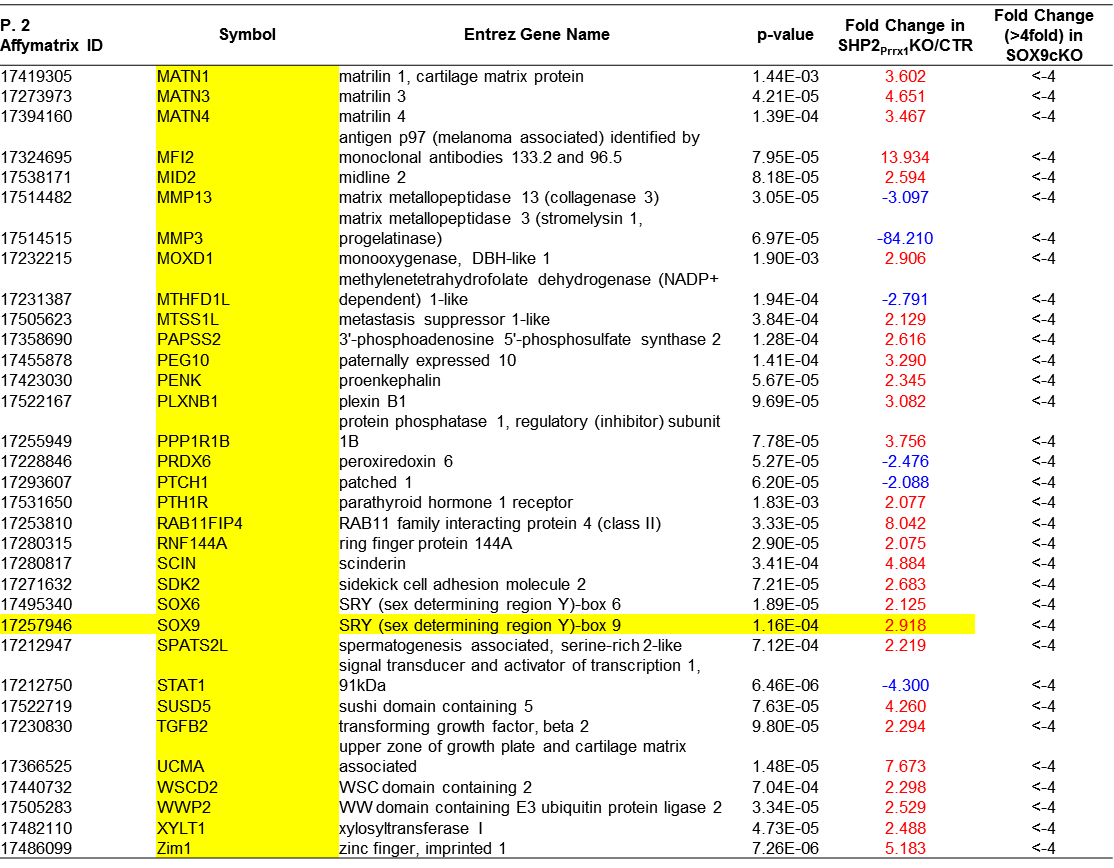


**
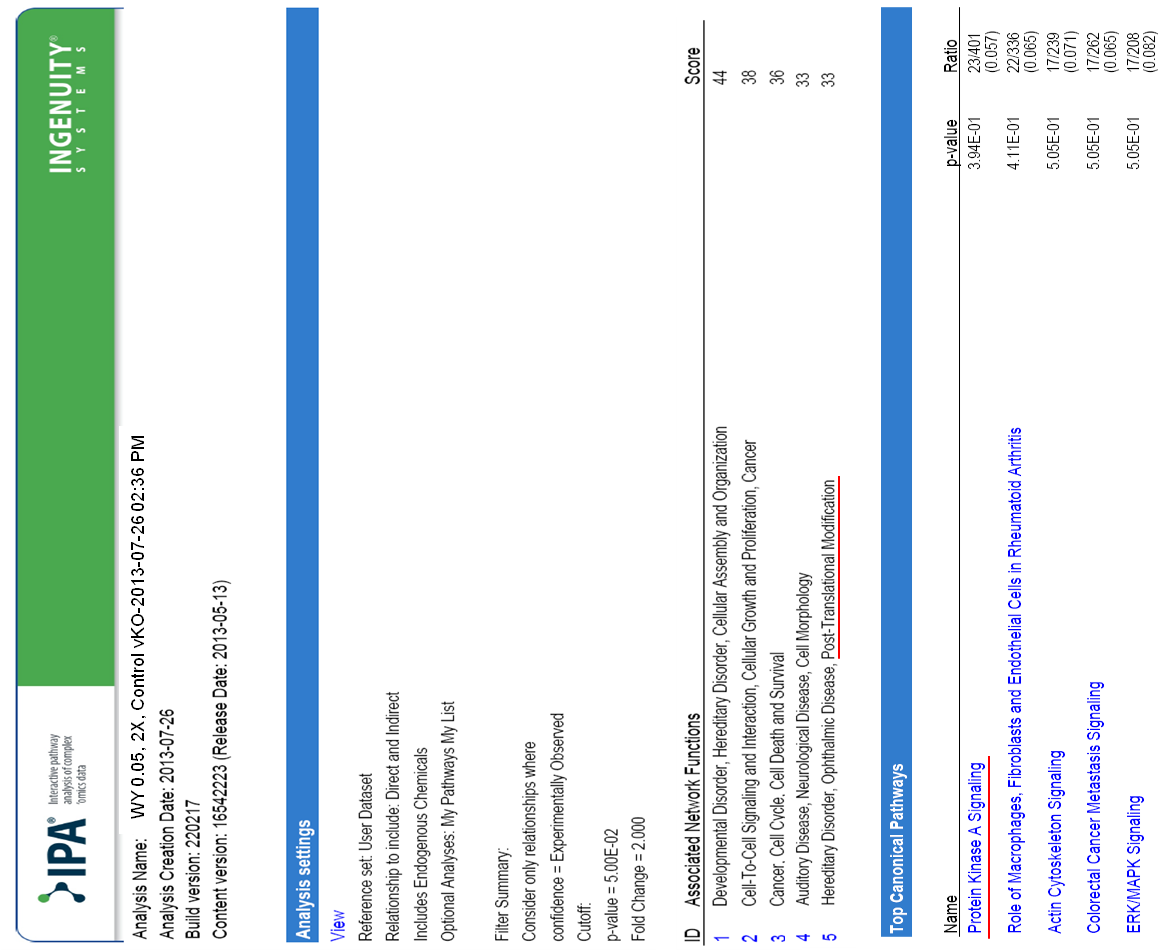
Figure S4 P3.** A list of selected chondrocytic genes that are up-regulated in OCPs and their derivatives from SHP2_Prrx1_KO;R26^mTmG^ mice are also down-regulated in the SOX9^-/-^ chondroid cells according to the published literature(see below*). Ingenuity pathway analysis predicting that PKA signaling pathway is mostly affected. *1. C. D. Oh, Y. Lu, S. Liang, Y. Mori-Akiyama, D. Chen, B. de Crombrugghe, H. Yasuda, SOX9 Regulates Multiple Genes in Chondrocytes, Including Genes Encoding ECM Proteins, ECM Modification Enzymes, Receptors, and Transporters. PloS one 9, e107577 (2014). *2. C. D. Oh, S. N. Maity, J. F. Lu, J. Zhang, S. Liang, F. Coustry, B. de Crombrugghe, H. Yasuda, Identification of SOX9 interaction sites in the genome of chondrocytes. PloS one 5, e10113 (2010).


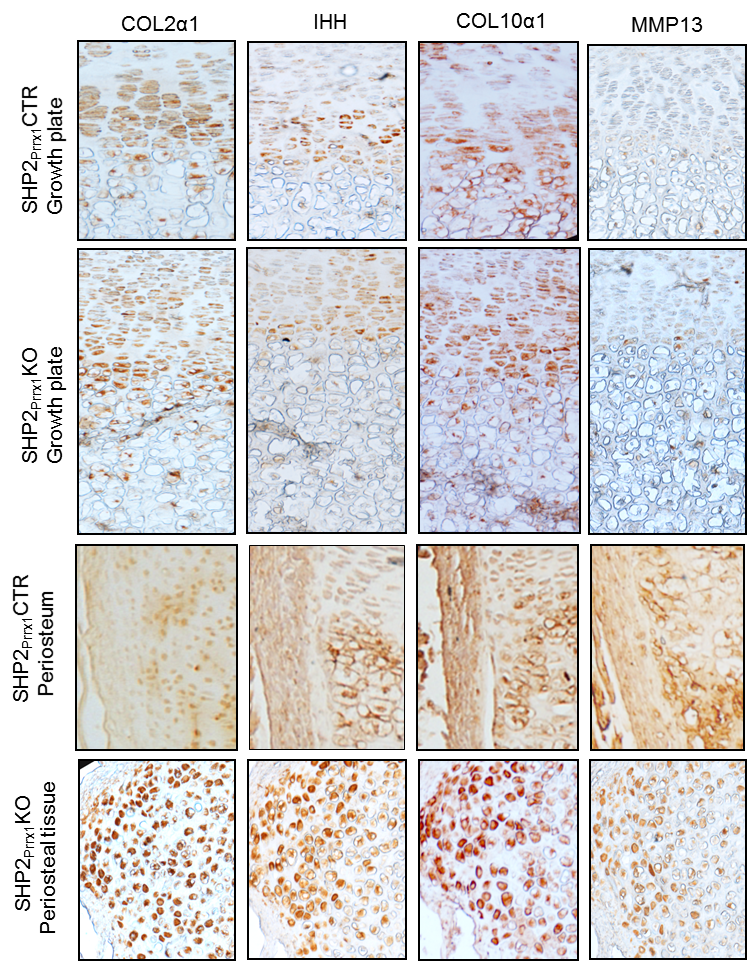


**Figure S5. SHP2 deficiency in OCPs leads to more widespread abundance of SOX9-associated cartilage proteins.** Representative images of proximal tibia sections of 7-day-old SHP2_Prrx1_CTR and SHP2_Prrx1_KO mice immunostained with antibodies against type II (COL2a1) and X (COL10a1) collagen, Indian hedgehog (IHH), and MMP13. Note the increase of SOX9-associated protein abundance and cells expressing COL2a1, COL10a1, and IHH. MMP13 abundance is elevated only in periosteal soft tissue of SHP2_Prrx1_KO but not SHP2_Prrx1_CTR mice (n=3).

**
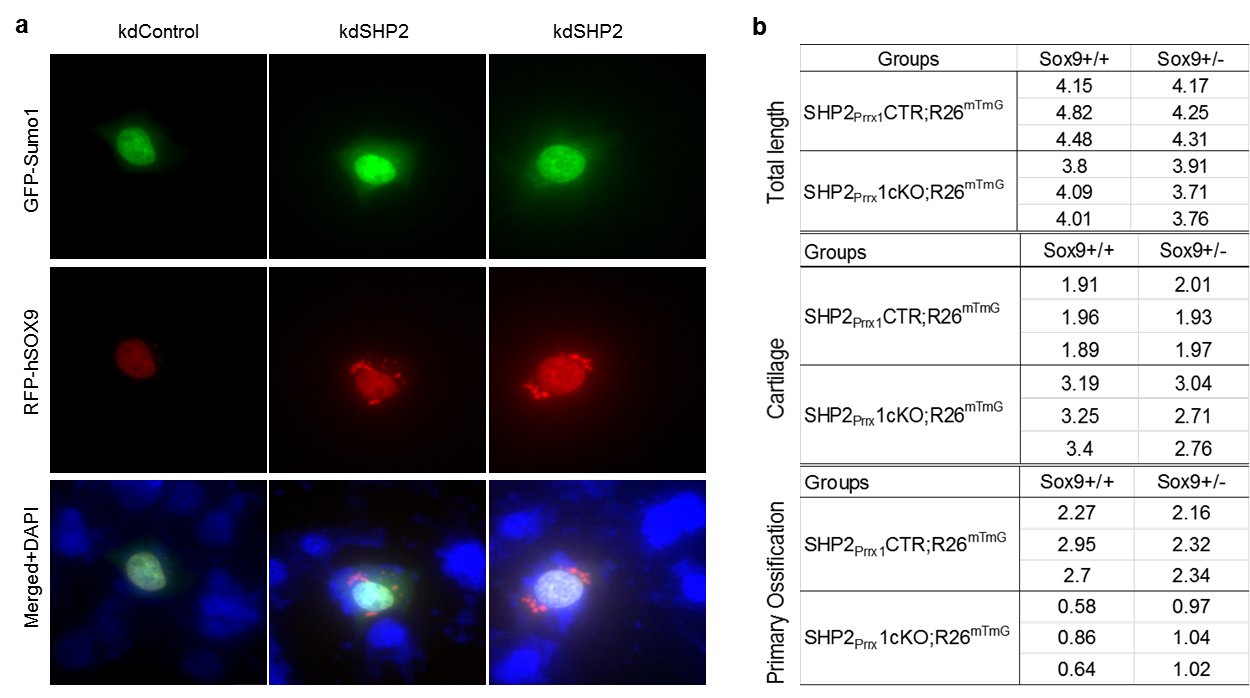
**

**Figure S6. a.** Knockdown of SHP2 expression in chondroprogenitors results in elevated SOX9 expression and protein SUMOylation. Control (kdControl) and SHP2 knockdown (kdSHP2) ATDC5 cells were co-transfected with GFP-tagged SUMO1 and RFP-tagged SOX9. Fluorescence microscopic images were taken 78 hours post-transfection to demonstrate the elevated SOX9 abundance (judged by GFP intensity) and protein SUMOylation (judged by RFP intensity). DAPI was used as a counterstaining to visualize transfected and untransfected cells. **b.** Quantitative data of Figure 6.


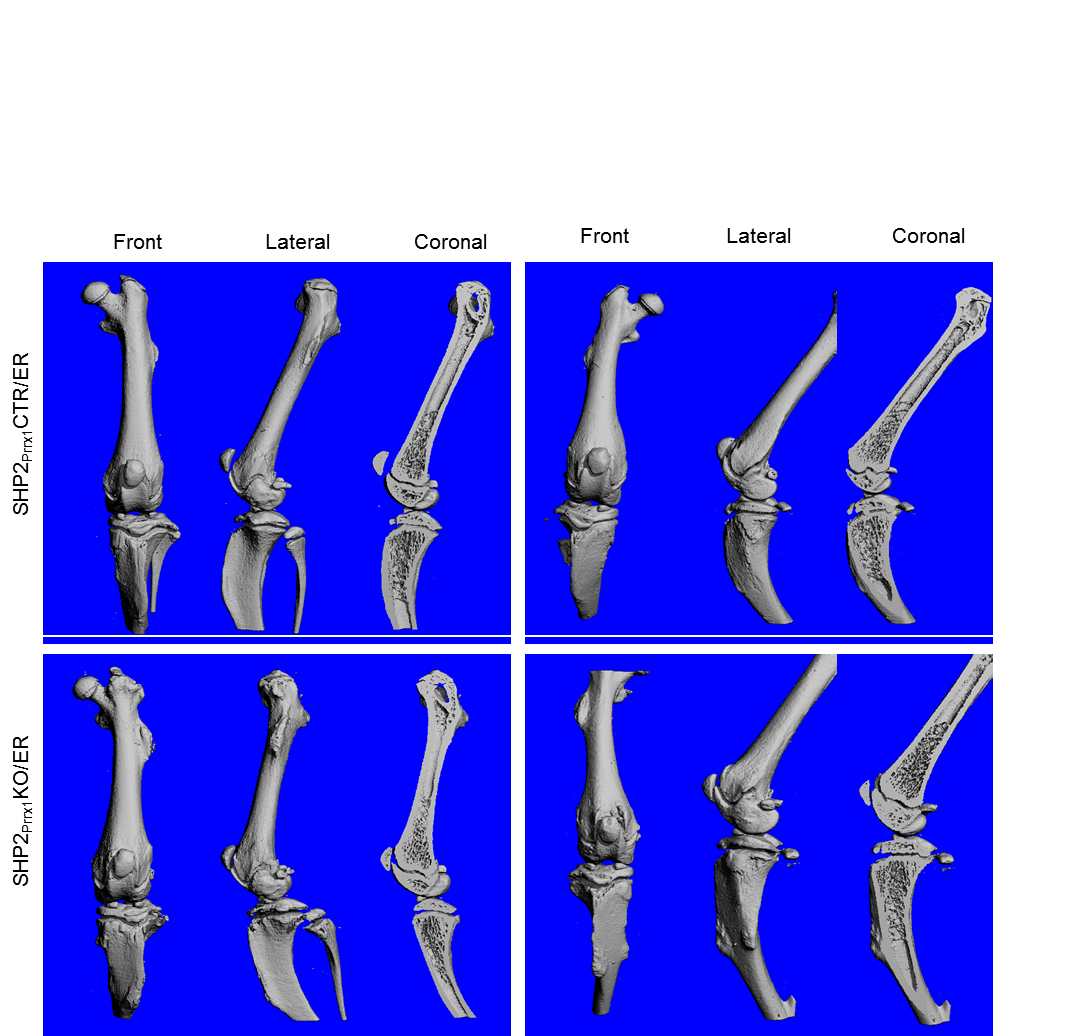


**Figure S7.** Micro-CT images of femora and tibiae from 12-week-old SHP2_Prrx1_CTR/ER and SHP2_Prrx1_KO/ER mice that had been administered 4-OH tamoxifen (0.1mg/per mouse) at postnatal week 2. Mice with mosaic SHP2 deletion in PRRX1-expressing OCPs had no detectable exostoses and enchondromas at this age (n=5 for each genotype).


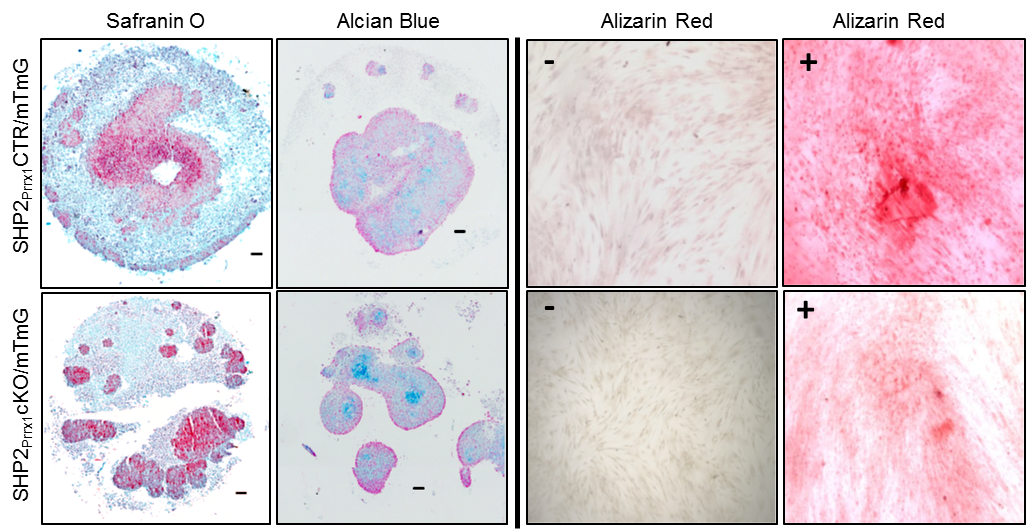


**Fig. S8** SHP2 deletion in OCPs enhances chondrogenic but inhibit osteogenic differentiation. **A.** 1x10^6^ of SHP2 sufficient and deficient OCPs were cultured in pellets in the chondrogenic medium for 12 days and stained with Safranin O/fast green and alcain blue/Safrinin O to visualize chongrogenesis. **B.** 6x10^6^ of SHP2 sufficient and deficient OCPs were cultured in osteogenic medium for 14 days and stained with alizarin red to visualize osteogenesis. Note that SHP2 deletion promotes chondrogenesis as reflected by enhanced SO and AB staining, but inhibit osteogenesis, judged on reduced alizarin red staining.


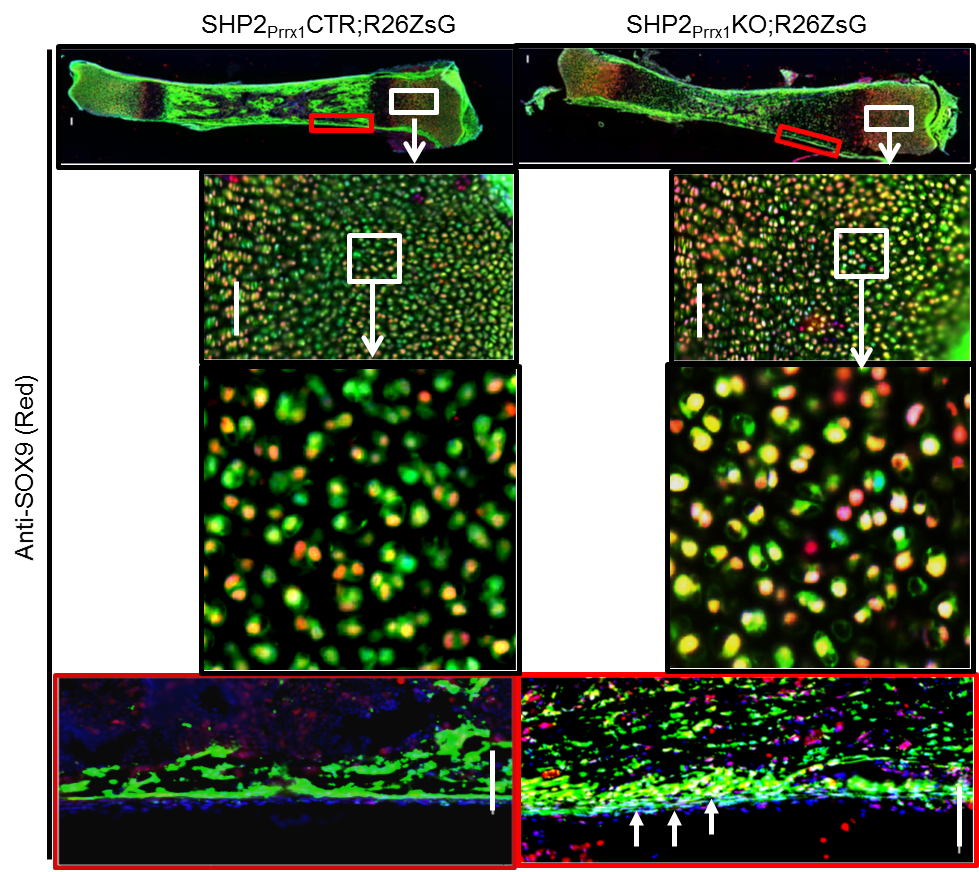


**Fig. S9** SHP2 deletion in PRRX1+ OCPs leads to more widespread abundance of SOX9. Images of frozen tibia section of 0.5-day-old mice immunostained with antibodies against SOX9 (red) demonstrate the elevated number of PRRX1+ SOX9+ cells in the periosteal (red line box) and epiphyseal area (white line box) in *SHP2_Prrx1_KO;R26^ZsG^* mice, compared to *SHP2_Prrx1_CTR;R26^ZsG^*. Scale bar:100µm.


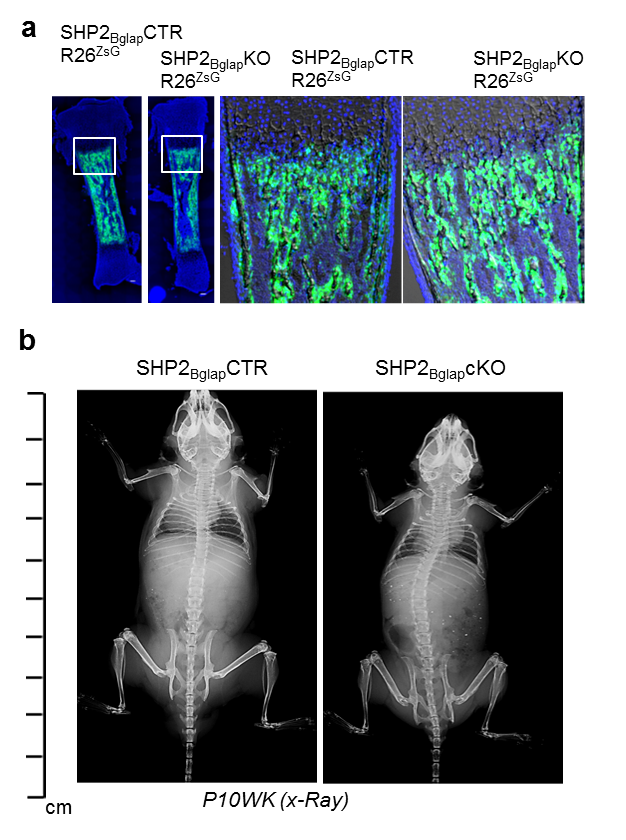


**Fig. S10** SHP2 deletion in BGLAP (Osteocalcin)+ osteoblasts has no apparent effect on trabecular and cortical bone formation at postnatal day P0.5 and on gross skeletal growth by 10 weeks age. **a.** Images of frozen tibia section of 0.5-day-old SHP2_Bglap_CTR;R26^ZsG^ and SHP2_Bglap_KO;R26^ZsG^ mice counterstained with DAPI (blue) demonstrate the number and distribution of GFP+ SHP sufficient and deficient osteoblasts in the developing tibia (left). Images on the right are enlarged view of the corresponding boxed areas on the left. n=5. **b.** Faxitron images show that SHP2_Bglap_CTR and SHP2_Bglap_KO mice have comparable skeletal morphology and mineralization at 10 weeks old. n=5.
